# Evaluating therapeutic potential of *NR2E3* doses in the *rd7* mouse model of retinal degeneration

**DOI:** 10.1038/s41598-024-67095-6

**Published:** 2024-07-17

**Authors:** Shannon M. McNamee, Monica Akula, Zoe Love, Neelaab Nasraty, Kaden Nystuen, Pushpendra Singh, Arun K. Upadhyay, Margaret M. DeAngelis, Neena B. Haider

**Affiliations:** 1 Schepens Eye Research Institute, Massachusetts Eye and Ear, Department of Ophthalmology, Harvard Medical School, 20 Staniford Street, Boston, MA 02114 USA; 2 University of Massachusetts Amherst, Amherst, MA USA; 3 Ocugen, Inc., Malvern, PA USA; 4 Department of Ophthalmology, Jacobs School of Medicine and Biomedical Sciences, University at Buffalo, Buffalo, NY USA; 5 Department of Cell Biology, Harvard Medical School, Boston, MA 02138 USA

**Keywords:** Gene replacement, AAV, Retinitis pigmentosa, rd7, Mouse, Electroretinography, Histology, Gene therapy, Retinal diseases, Preclinical research

## Abstract

Retinitis Pigmentosa is a leading cause of severe vision loss. Retinitis Pigmentosa can present with a broad range of phenotypes impacted by disease age of onset, severity, and progression. This variation is influenced both by different gene mutations as well as unique variants within the same gene. Mutations in the nuclear hormone receptor 2 family e, member 3 are associated with several forms of retinal degeneration, including Retinitis Pigmentosa. In our previous studies we demonstrated that subretinal administration of one *Nr2e3* dose attenuated retinal degeneration in *rd7* mice for at least 3 months. Here we expand the studies to evaluate the efficacy and longitudinal impact of the *NR2E3* therapeutic by examining three different doses administered at early or intermediate stages of retinal degeneration in the *rd7* mice. Our study revealed retinal morphology was significantly improved 6 months post for all doses in the early-stage treatment groups and for the low and mid doses in the intermediate stage treatment groups. Similarly, photoreceptor function was significantly improved in the early stage for all doses and intermediate stage low and mid dose groups 6 months post treatment. This study demonstrated efficacy in multiple doses of *NR2E3* therapy.

## Introduction

Retinitis Pigmentosa (RP) is a heterogeneous, pleiotropic group of inherited retinal diseases which affect every 1 in 4000 people and occur in syndromic and non-syndromic forms^1–10^. RP can occur through multiple modes of inheritance including autosomal-dominant (15–25% of cases), autosomal-recessive (5–20% of cases), X-linked (5–15% of cases), and simplex (40–50% of cases)^2,6,7,11,12^. Non-syndromic and syndromic forms of RP are associated with over 4300 mutations across more than 100 identified genes resulting in variable age of onset, disease progression rate, and severity depending on the specific mutations and genes impacted^1,12–14^. Despite the considerable phenotypic variation in RP, photoreceptor cell degeneration is the primary pathology identified in all types of RP. Currently, there is no cure for RP and the disease is genetically unidentifiable in approximately 50% of patients^11,15^.

In recent studies our lab demonstrated the novel use of nuclear hormone receptor 2 family e, member 3 (*Nr2e3*) as a genetic modifier and therapeutic for several forms of RP in multiple mouse models including *rd7*^16^. Treatment of each RP mouse model with a subretinal injection of *Nr2e3* resulted in stabilized retinal function, preserved retinal morphology, increased photoreceptor survival, and altered expression in several genes and transcription factors associated with biological pathways involved in maintaining retinal homeostasis and development. The study demonstrated improvements at the functional, histological, and immunohistochemical level of 30–80% in all RP models. *Nr2e3* was shown to be expressed in the regions of rescue in treated retinas. The study by Li et al*.* also investigated the mechanism of *Nr2e3* rescue via changes in gene expression in 8 *Nr2e3*-directed networks involved in maintaining retinal homeostasis: apoptosis, ER stress, immunity, metabolism, neuroprotection, oxidative stress, phototransduction, and cell survival. Several genes in each of these networks were differentially expressed following *Nr2e3* treatment in *rd7* mice indicating the involvement of *Nr2e3* in modulating networks involved in retinal homeostasis^16^. Our present study builds on the findings of Li et al*.* by examining efficacy of three different *NR2E3* GLP quality doses, extending the longitudinal observation time to 6 months after *NR2E3* administration, and examining treatment during early and intermediate stages of disease progression in order to inform clinical trials.

*NR2E3* is a nuclear hormone receptor expressed in retinal progenitors, rods, and cones^17,18^, and as such plays a pivotal role in modulating several biological pathways essential for maintaining homeostasis in the retina including apoptosis, cell survival, ER stress, immunity, metabolism, neuroprotection, oxidative stress, and phototransduction^16^. *NR2E3* associated retinal diseases include several recessive diseases: clumped pigmentary retinal degeneration (CPRD), enhanced S-cone syndrome (ESCS), Goldmann–Favre syndrome (GFS), and autosomal dominant Retinitis Pigmentosa (adRP)^19–27^. GFS and CPRD patients with *NR2E3* mutations share some clinical phenotypes common in ESCS such as night blindness, reduced rod function, and hyper S-cone function^25,28^. ESCS in humans is characterized by an increase in S (short, blue) cone photoreceptor function along with degeneration in rods and red and green cones that results in blue light sensitivity, night blindness and vision loss^23,29–34^. GFS is a vitreoretinal degenerative disease, considered to potentially be a milder form of ESCS, that presents with cystoid macular edema (CME), foveal and peripheral retinoschisis, night blindness, pigmentary degeneration, and vitreous changes^28,35,36^. CPRD is characterized by typical RP symptoms accompanied by accumulation of melanin granules in the retinal pigment epithelium (RPE) resulting in large pigment deposits in the peripheral retina^25,37,38^. The correlation of *NR2E3* with various disease phenotypes and modes of inheritance strongly suggests that these degenerative diseases likely develop on a permissive or selective genetic background and are likely influenced by the specific mutations as well as modifier genes^16,20,22,24,39^.

Adeno-associated viruses (AAVs) have proven to be effective therapeutic gene delivery vectors for treating diseases such as inherited retinal degeneration due to their variability in cell type specificity, long term expression and low immune response^40–47^. Adeno-associated virus serotype 5 (AAV5) containing the human *NR2E3* gene (AAV5-*hNR2E3*) was chosen as the viral vector for this study based on our previous study that examined AAV2.7m8, AAV5, and AAV8 gene delivery^16^ along with several safety and efficacy studies^40,41,44^. AAV5 transduces RPE and photoreceptor cells more efficiently and in greater volumes than other AAVs, especially AAV2^40–42^. Studies also showed that AAV5 has a low immunogenic response in humans due to their low numbers of pre-existing circulating AAV5 antibodies^44^.

This is a pharmacological preclinical longitudinal study of the dose–response and efficacy of subretinal delivery of AAV5-*hNR2E3* to ameliorate and attenuate retinal degeneration when administered during early (postnatal day (P) 30) or intermediate (3-months of age) disease progression in the RP mouse model *rd7* 1, 3, and 6 months after treatment. The *Nr2e3*^*rd7*^*/J* (*rd7*) mouse is a model for recessive *NR2E3*-associated retinal disease^18,48–50^. The *rd7* mouse exhibits bilateral pan retinal spots observed at eye opening (P14) which fade with degeneration, and whorls and rosettes in the outer nuclear layer (ONL) of the retina that are apparent histologically by P10^16,18,50^. As the phenotype progresses, a flattening of whorls and reduction of retinal spots is observed around 5 months of age followed by progressive loss of the ONL and outer segment thickness and appearance of mottled retinal pigment by 16 months of age^50^. Also, electroretinogram levels do not fall below normal until about 5 months of age and are reduced by approximately 50% by 16 months of age^50^. There are two components of disease in *rd7* mice: a developmental problem in the retinal progenitors resulting in abnormal increase in blue opsin expressing cone cells due to over proliferation of cone cells (not at the expense of other cell types such as rods), followed by a slow and progressive degeneration of cone and rod photoreceptor cells^18,50^. The results of this study showed that administration of AAV5-*hNR2E3* at any of the three doses evaluated resulted in sustained attenuation of retinal degeneration over 6 months when administered during early or intermediate progression of *rd7* retinal degeneration.

## Results

### Subretinal delivery of three doses of AAV5-*hNR2E3* in *rd7* mice during early or intermediate disease stages shows no adverse effects on the clinical phenotype

**Figure 1:**
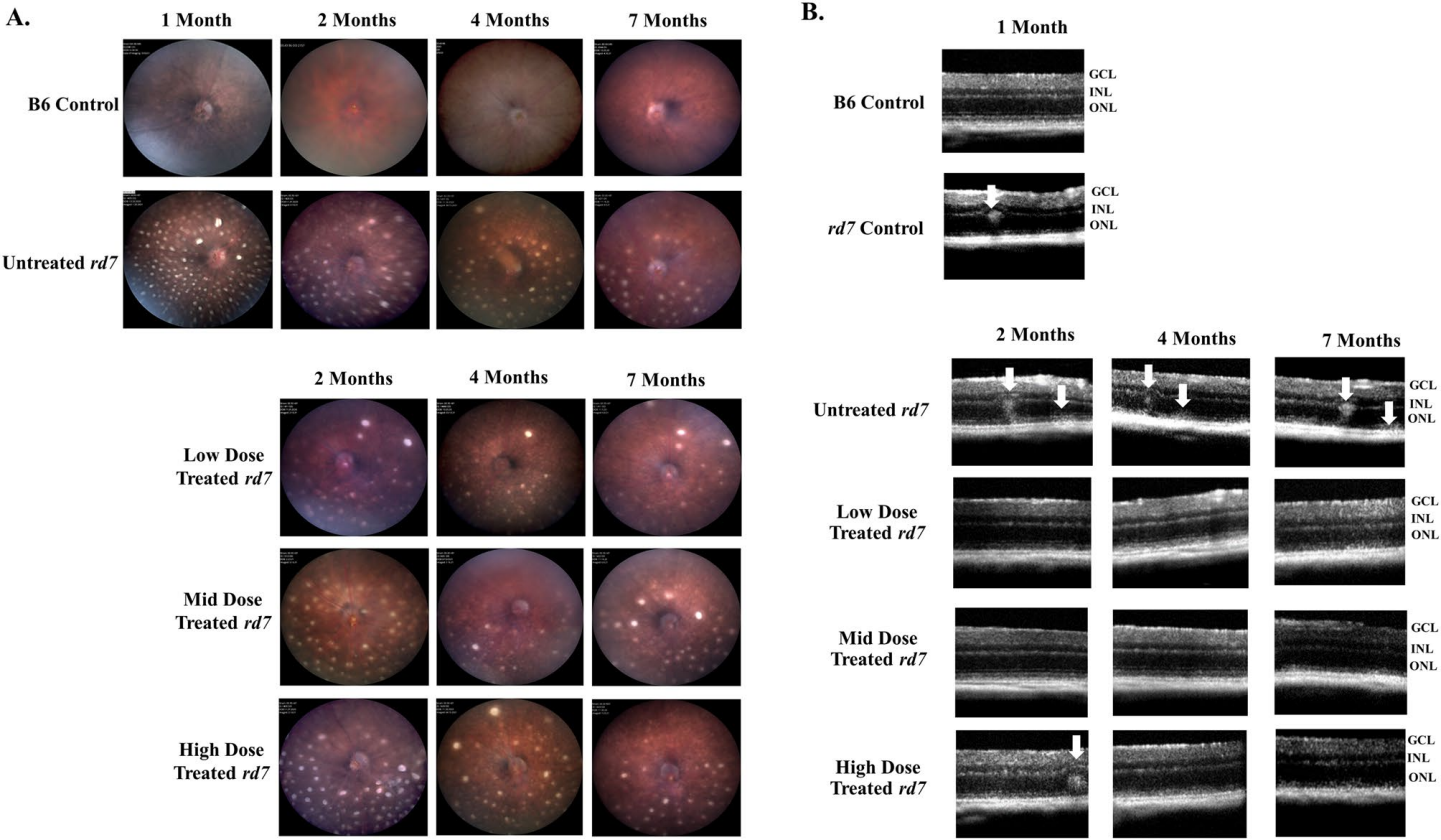
Treatment during early disease in *rd7* mice with low, mid, and high dose *NR2E3* showed no adverse effects on the clinical phenotype. (**A**) Fundus of C57BL6/J (B6) wild-type and untreated *rd7* animals used for pre-treatment and age matched controls, and (**B**) *rd7* animals injected at P30 with low, mid, and high dose *NR2E3* and assessed 1-, 3-, and 6-months post injection (animal ages 2, 4, and 7 months). (**B**) Optical coherence tomography of 1 month old wild-type C57BL6/J (B6), and untreated *rd7* mice as pre-treatment controls (upper panels). *rd7* animals dosed at P30 and assessed 1-, 3-, and 6-months post injection (animal ages 2, 4, and 7 months), and age matched untreated *rd7* controls (lower panels). Arrows indicate whorls and rosettes. All images were collected at approximately the same location at the central retina. Low Dose = 1 × 10^8^ v/gc, Mid Dose = 1 × 10^9^ v/gc, and High Dose = 4 × 10^9^ v/gc. n ≥ 5.

**Figure 2:**
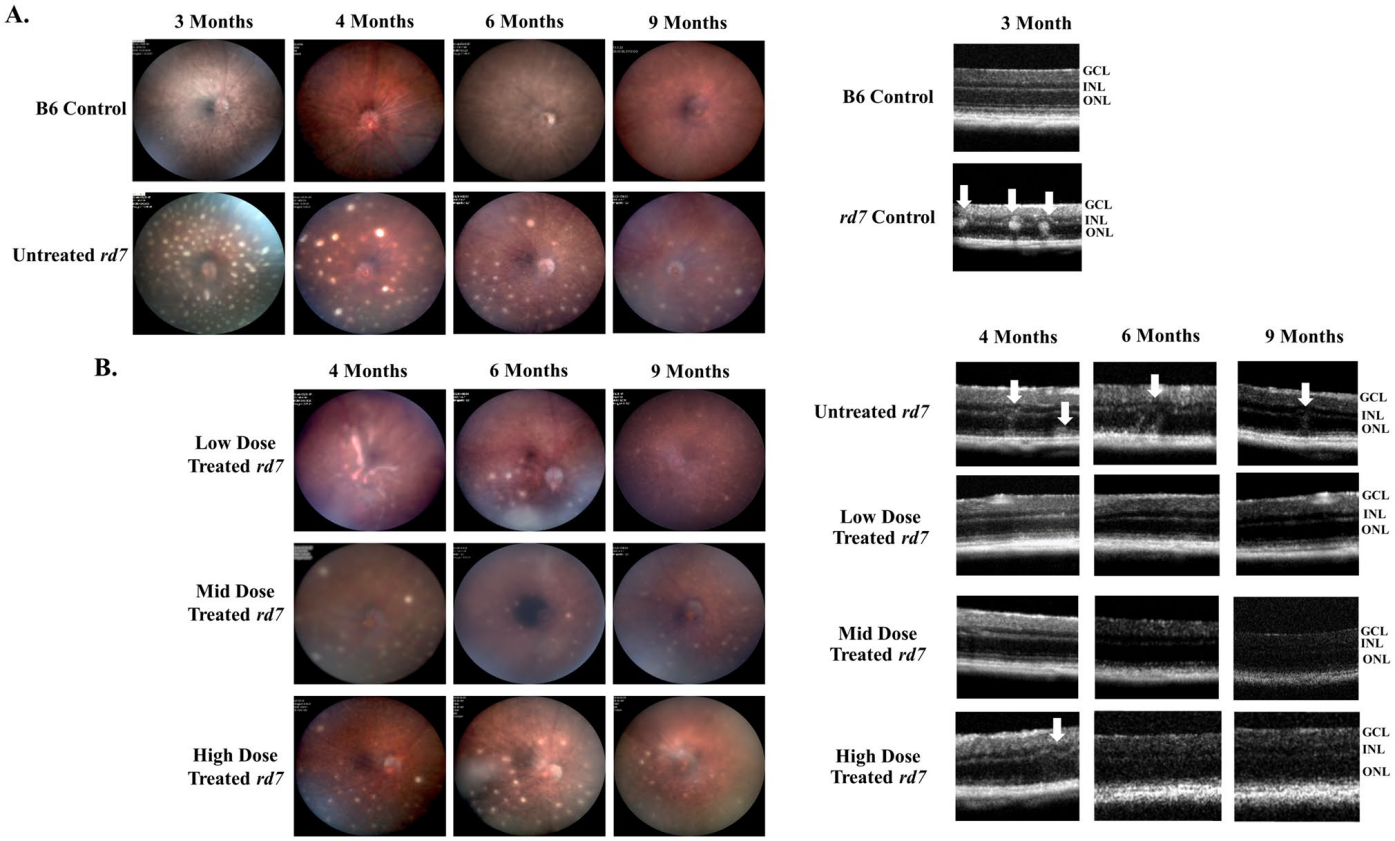
Low, mid, and high dose *NR2E3* treatment in *rd7* mice during intermediate degeneration revealed no adverse effects on clinical phenotype. (**A**) Fundus of C57BL6/J (B6) wild-type and untreated *rd7* animals used for pre-treatment and age matched controls, and (**B**) *rd7* animals injected at 3-months of age with low, mid, or high dose *NR2E3* and assessed 1-, 3-, and 6-months post injection (animal ages 4, 6, and 9 months). (**B**) Optical coherence tomography of (**A**). 3-month-old wild-type C57BL6/J (B6), and untreated *rd7* mice as pre-treatment controls (upper panels). *rd7* animals dosed at 3-months of age and assessed 1-, 3-, and 6-months post injection (animal ages 4, 6, and 9 months), and age matched untreated *rd7* controls (lower panels). Arrows indicate whorls and rosettes. All images were collected at approximately the same location at the central retina. Low Dose = 1 × 10^8^ v/gc, Mid Dose = 1 × 10^9^ v/gc, and High Dose = 4 × 10^9^ v/gc. n ≥ 5.

The long-term effects of *NR2E3* on the clinical phenotype of *rd7* animals were evaluated following subretinal delivery of AAV5 during early or intermediate stages of *rd7* clinical disease progression. Animals were treated with either a low, mid, or high dose of AAV5-*hNR2E3* at P30 or 3 months of age, and evaluated 1-, 3-, and 6-months post treatment to monitor for any longitudinal adverse effects of *NR2E3* on the *rd7* animals. The *rd7* fundus phenotype has pan retinal spots that lack a consistent spatial or numerical pattern; with variability in the density of spots. These spots fade as degeneration progresses; thus, while it is not useful to quantify the number of retinal spots, the fundus can be used to qualitatively evaluate potential signs of gross abnormalities or inflammation due to treatment, such as retinal scarring, by comparing treated eyes with uninjected and mock injected eyes. Early-stage fundus analysis of *rd7* animals treated with *NR2E3* at P30 revealed no long-term negative effects such as an increase in pan retinal spots or inflammation in the fundus phenotype for all doses (Fig. 1). Similarly, longitudinal monitoring of intermediate stage treatment (*rd7* animals treated at 3-months of age) with *NR2E3* revealed no gross abnormalities in the fundus phenotype for all three therapeutic doses (Fig. 2). OCT was performed to track in life observation of any potential adverse effects. No adverse effects were observed in 1-, 3-, and 6-month post early stage (Fig. 1B) and intermediate stage (Fig. 2B) *NR2E3* treated *rd7* mice. Microglial cells are the main constituents of the immune cell population in the retina and the main initiators of the inflammatory response in the retina^51–54^. Our prior studies showed that *rd7* mice do not undergo inflammation^17^. In order to determine if AAV5-*hNR2E3* treatment will induce an inflammatory response through microglial activation, we examined the expression of IBA1, a marker of microglial activation. The results show that there is no evidence of microglial activation reflecting absence of inflammation in *NR2E3*-treated *rd7* mice, similar to normal B6 and untreated *rd7* mice (Supplemental Fig. S1).

### AAV delivery of *NR2E3* in different doses in *rd7* animals during early or intermediate disease progression restores retinal morphology

**Figure 3:**
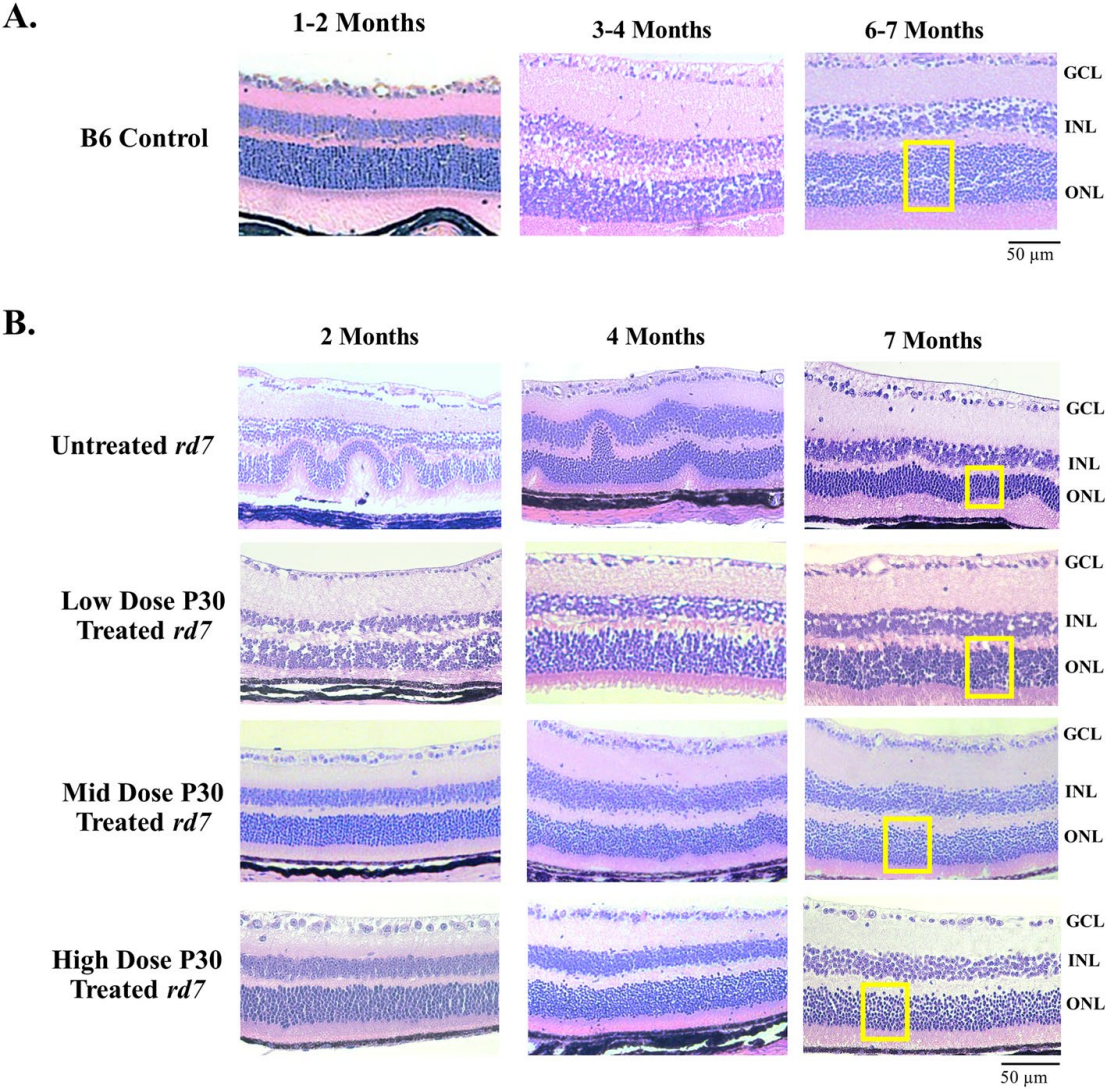
AAV5-*hNR2E3* treatment at P30 rescues retinal morphology in *rd7* retinas in low, mid, and high doses. (**A**) C57BL6/J (B6) 1-, 3-, and 6-month wild-type H/E. (**B**) *rd7* animals injected at P30 and collected 1-, 3-, and 6-months post injection (animal ages 2, 4, and 7 months) with low, mid, or high dose therapy, and age matched untreated *rd7* controls. White boxes on the 7-month images indicate where cell counts were performed representatively. Low Dose = 1 × 10^8^ v/gc; Mid Dose = 1 × 10^9^ v/gc, High Dose = 4 × 10^9^ v/gc. *GCL* Ganglion Cell Layer, *INL* Inner Nuclear Layer, *ONL* Out Nuclear Layer. Scale bar = 50 µm. n ≥ 5.

**Figure 4:**
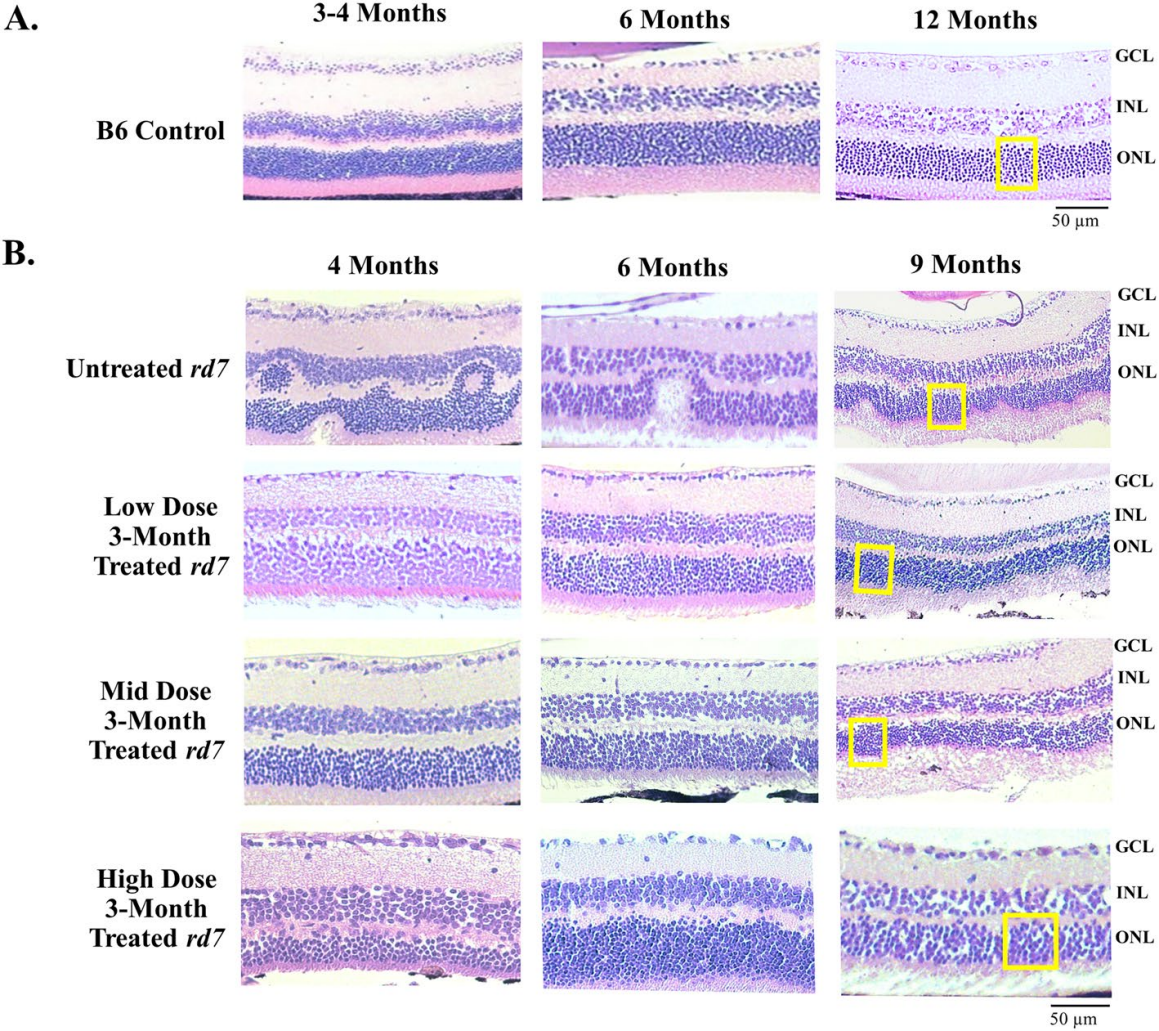
AAV5-*hNR2E3* treatment during intermediate disease rescues retinal morphology in *rd7* retinas in low, mid, and high doses. (**A**) C57BL6/J (B6) 3-, 6-, and 12-month wild-type H/E. (**B**) *rd7* animals injected at 3-months of age and collected 1-, 3-, and 6-months post injection (animal ages 4, 6, and 9 months) with low, mid, or high dose therapy, and age matched untreated *rd7* controls. White boxes on the 9-month images indicate where cell counts were performed representatively. Low Dose = 1 × 10^8^ v/gc; Mid Dose = 1 × 10^9^ v/gc, High Dose = 4 × 10^9^ v/gc. *GCL* Ganglion Cell Layer, *INL* Inner Nuclear Layer, *ONL* Out Nuclear Layer. Scale bar = 50 µm. n ≥ 5.

**Figure 5:**
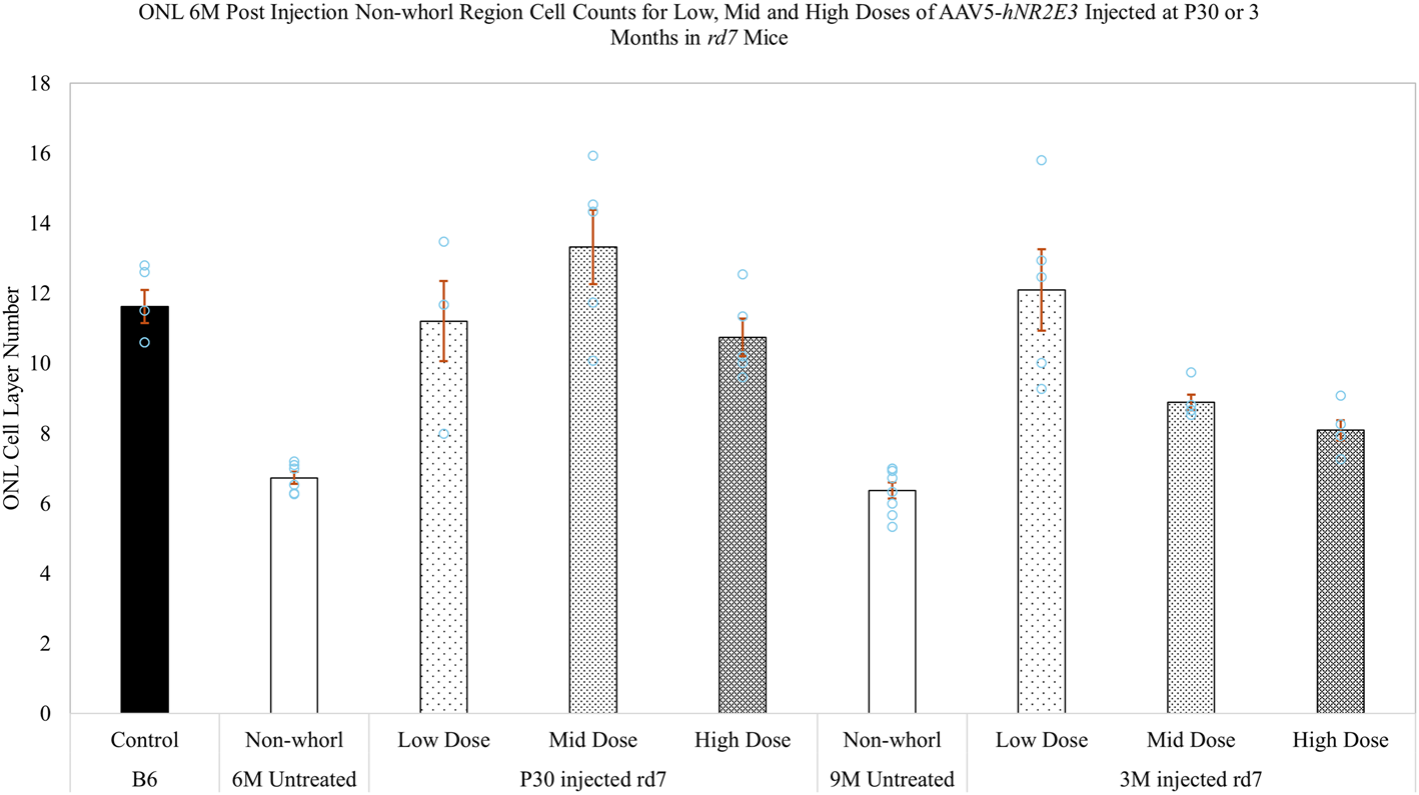
AAV5-*hNR2E3* treatment reduces ONL degeneration from disease progression in *rd7* animals at all doses. Outer nuclear layer (ONL) thickness of animals injected at P30 (early) or 3-months of age (intermediate) and collected 6-months post injection (animal ages 7 months and 9 months) were compared with the non-whorl region of untreated *rd7* animals of the same age. Early AAV5-*hNR2E3*-treated *rd7* animals untreated vs low dose p < 0.006, untreated vs mid dose p < 0.0001, untreated vs high dose p < 0.009. Intermediate AAV5-*hNR2E3*-treated *rd7* animals untreated vs low dose p < 0.0001, untreated vs mid dose p < 0.04; low dose vs mid dose p < 0.02; low dose vs high dose p < 0.002. Dots represent individual data points for each experimental group. Serial section of central retina counted over 100 µm. Low Dose = 1 × 10^8^ v/gc; Mid Dose = 1 × 10^9^ v/gc, High Dose = 4 × 10^9^ v/gc. Results are mean ± SEM, n ≥ 5.

Histology analysis of *rd7* animals dosed with AAV5-*hNR2E3* during early or intermediate disease progression showed improved retinal morphology. *rd7* mice exhibit a slow, progressive, concomitant degeneration of rod and cone photoreceptors, leading to resolution of the whorls over time. A normal mouse retina consists of 10–12 layers of cone and rod photoreceptor nuclei making up the ONL and 5–6 layers of inner retinal cells making up the inner nuclear layer (INL)^16^. In the *rd7* model, abnormal morphology presents with whorls and rosettes in the ONL caused by over-proliferation of blue opsin expressing cone cells followed by slow, progressive photoreceptor degeneration^18,50^. The whorls and rosettes begin to resolve and flatten by 5 months of age as degeneration progresses^50^. The non-whorl regions of the retina are the most important for tracking degeneration and rescue as abnormal blue cone proliferation has not disrupted the normal thickness of this region prior to disease onset. Hematoxylin/Eosin (H/E) staining showed fewer whorls and rosettes and increased ONL thickness in P30 treated retinas for all doses over 6 months compared to untreated retinas (Fig. 3, lower magnification in Supplemental Fig. S2). H/E analysis of *rd7* animals treated with *NR2E3* at 3-months of age similarly demonstrated a reduction in whorls and rosettes and increased ONL thickness in treated retinas for all doses compared to untreated maintained over 6 months (Fig. 4). Some whorls and rosettes will still be present in treated retinas if they were present prior to *NR2E3* administration halting proliferation and degeneration. Additionally, to discern the impact of rescue, the ONL in the non-whorl and rosette regions of *rd7* animals treated at P30 or 3-months of age and collected 6-months after treatment were counted and compared with untreated retinas. Statistically significant rescue of photoreceptor cells in the non-whorl regions was observed compared to untreated animals in both early and intermediate administration groups except for the intermediate stage high dose treatment (Fig. 5; *p* < 0.05 to 0.0001). Both the P30 and 3-month injected low dose groups rescued similar amounts of photoreceptors. Interestingly, animals treated at P30 showed rescue to normal ONL thickness (around 10–12 layers) for all doses, compared to mid and high dose animals treated at 3-months of age that showed a more modest rescue of around 8–9 layers. This is likely due to degeneration already occurring when treatment was administered at the intermediate progression phase (3-months of age) and thus fewer cells remained that could be rescued. These data suggest that *NR2E3* is effective at halting degeneration when administered at early or a later stage of disease for all doses except the high dose later stage, and for at minimum 6 months after treatment.

### Cone and rod opsin expression is preserved in *rd7* animals treated during early or intermediate stage progression with AAV5-*hNR2E3* dose therapy

**Figure 6:**
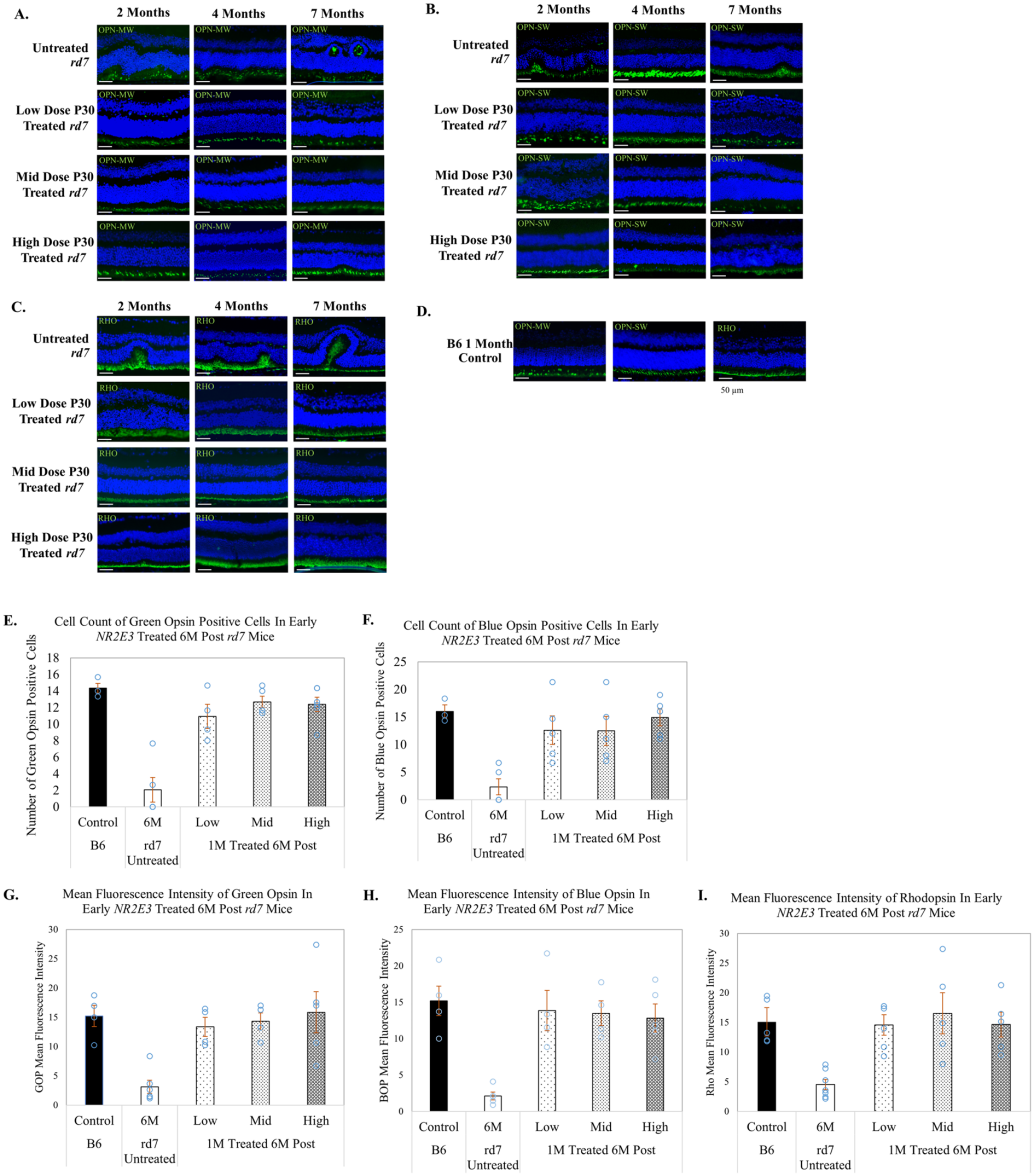
AAV5-*hNR2E3* treatment during early disease preserves opsin expression and restores retinal morphology in *rd7* mice at low, mid, and high doses. Immunohistochemical analysis of opsin expression in *rd7* animals treated with low, mid, and high dose *NR2E3* at P30 and collected 1-, 3-, and 6-months post treatment (animal ages 2, 4, and 7 months). (**A**) Green opsin (OPN-MW), (**B**) blue opsin (OPN-SW), and (**C**) rhodopsin (RHO) expression in treated and untreated animals for all doses and collection times. (**D**) C57BL6/J (B6) wild-type green opsin, blue opsin, and rhodopsin controls. (**E**,**F**) Green and blue opsin-positive cell counts in early *NR2E3* treated 6 months post *rd7* mice. Green opsin counts untreated vs low dose p < 0.0006, untreated vs mid dose p < 0.0001, untreated vs high dose p < 0.0001. Blue opsin counts untreated vs low and mid doses p < 0.04, untreated vs high dose p < 0.007. (**G**–**I**) Mean fluorescence intensity of green opsin, blue opsin, and rhodopsin in early *NR2E3*-treated 6 months post *rd7* mice. Green opsin intensity untreated vs low dose p < 0.05, untreated vs mid dose p < 0.03, untreated vs high dose p < 0.005. Blue opsin intensity untreated vs low dose p < 0.007, untreated vs mid and high doses p < 0.009. Rhodopsin intensity untreated vs low dose p < 0.03, untreated vs mid dose p < 0.005, untreated high dose p < 0.03. DAPI staining depicted in blue. Opsin shown in green. Low Dose = 1 × 10^8^ v/gc; Mid Dose = 1 × 10^9^ v/gc, High Dose = 4 × 10^9^ v/gc. Scale bar = 50 µm. Results are mean ± SEM, n ≥ 5.

**Figure 7:**
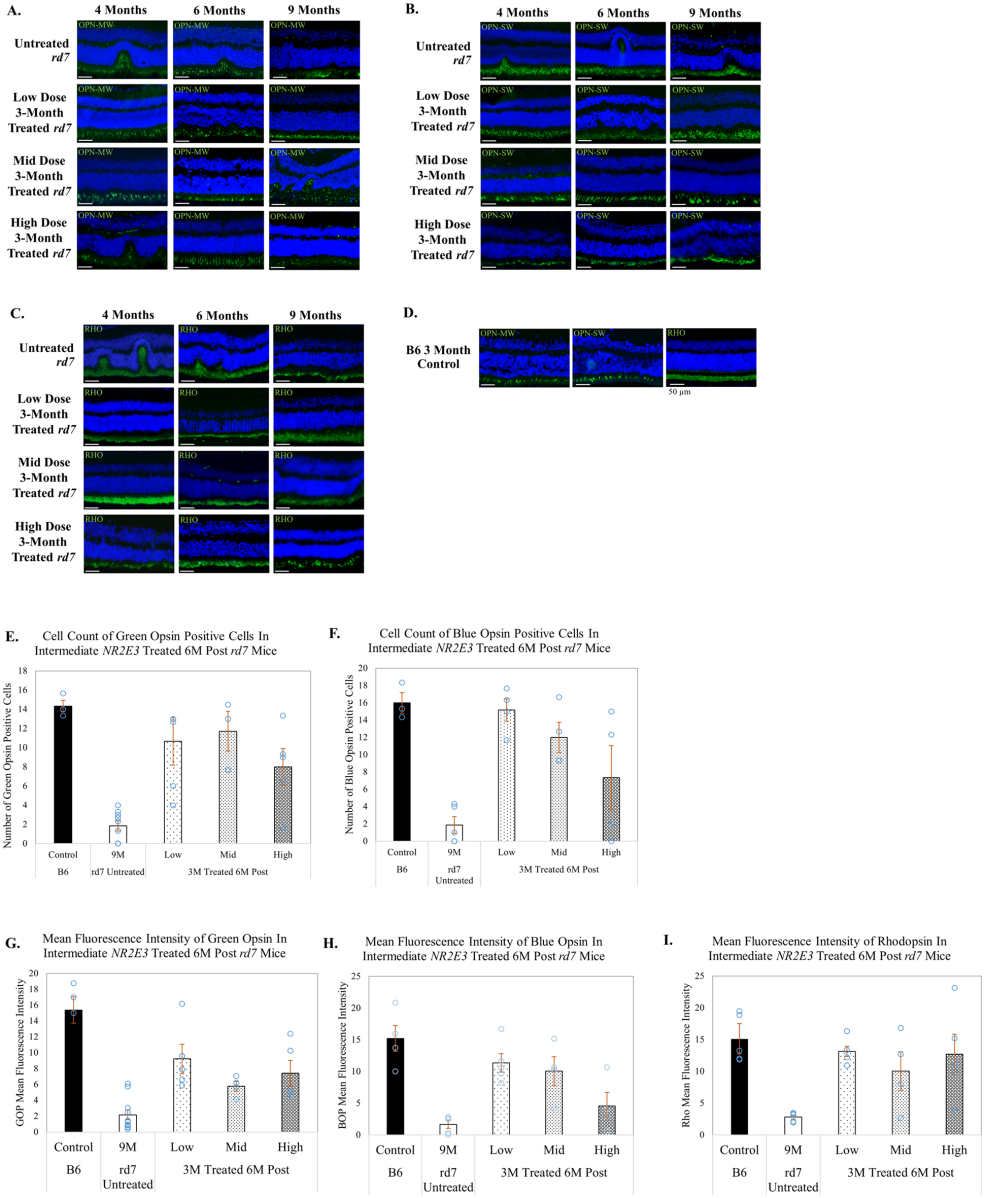
AAV5-*hNR2E3* treatment during intermediate disease preserves opsin expression and restores retinal morphology in *rd7* mice at low, mid, and high doses. Immunohistochemical analysis of opsin expression in *rd7* animals treated with low, mid, and high dose *NR2E3* at 3-months of age and collected 1-, 3-, and 6-months post treatment (animal ages 4, 6, and 9 months). (**A**) Green opsin (OPN-MW), (**B**) blue opsin (OPN-SW), and (**C**) rhodopsin (RHO) expression in treated and untreated animals for all doses and collection times. (**D**) C57BL6/J (B6) wild-type green opsin, blue opsin, and rhodopsin controls. (**E**,**F**) Green opsin-positive cell counts and blue opsin-positive cell counts in intermediate *NR2E3* treated 6 months post *rd7* mice. Green opsin counts untreated vs low dose p < 0.004, untreated vs mid dose p < 0.007. Blue opsin counts untreated vs low dose p < 0.004, untreated vs mid dose p < 0.04. (**G**–**I**) Mean fluorescence intensity of green opsin, blue opsin, and rhodopsin in intermediate *NR2E3*-treated 6 months post *rd7* mice. Green opsin intensity untreated vs low dose p < 0.02, untreated vs high dose p < 0.05. Blue opsin intensity untreated vs low dose p < 0.009, untreated vs mid dose p < 0.05. Rhodopsin intensity untreated vs low dose p < 0.02, untreated vs high dose p < 0.04. DAPI staining depicted in blue. Opsin shown in green. Low Dose = 1 × 10^8^ v/gc; Mid Dose = 1 × 10^9^ v/gc, High Dose = 4 × 10^9^ v/gc. Scale bar = 50 µm. Results are mean ± SEM, n ≥ 5.

Immunohistochemical analysis was performed to examine the efficacy of three different doses of AAV5-*hNR2E3* to evaluate blue and green cone opsin and rhodopsin expression in *rd7* mice. Our previous studies demonstrated that untreated *rd7* animals exhibit an increase in blue opsin expression followed by slow progressive loss of blue and green opsin and rhodopsin expression with disease progression over 5–16 months^16,18^. Animals treated with AAV5-*hNR2E3* at P30 showed preserved expression of all opsins compared to untreated animals for the low, mid, and high doses that was sustained to 6-months post treatment, assessed using green and blue opsin-positive cell counts and mean fluorescence intensity of the three opsins (p < 0.05 for all doses) (Fig. 6). Similarly, *rd7* mice treated with *NR2E3* at 3-months of age had preserved opsin expression sustained over 6 months after treatment for all doses, which was assessed using opsin cell counts and mean fluorescence intensity of the opsins (p < 0.05 for the low and mid doses for opsin counts) (Fig. 7). IHC analysis also supports our histological findings of partial rescue and restored retinal morphology from focal delivery of *NR2E3* dose treatment. All three doses showed rescue with no gross abnormalities or remarkable loss of expression of rod or cone opsins.

### AAV5-*hNR2E3* improved scotopic ERG responses in *rd7* animals treated during early or intermediate progression

**Figure 8:**
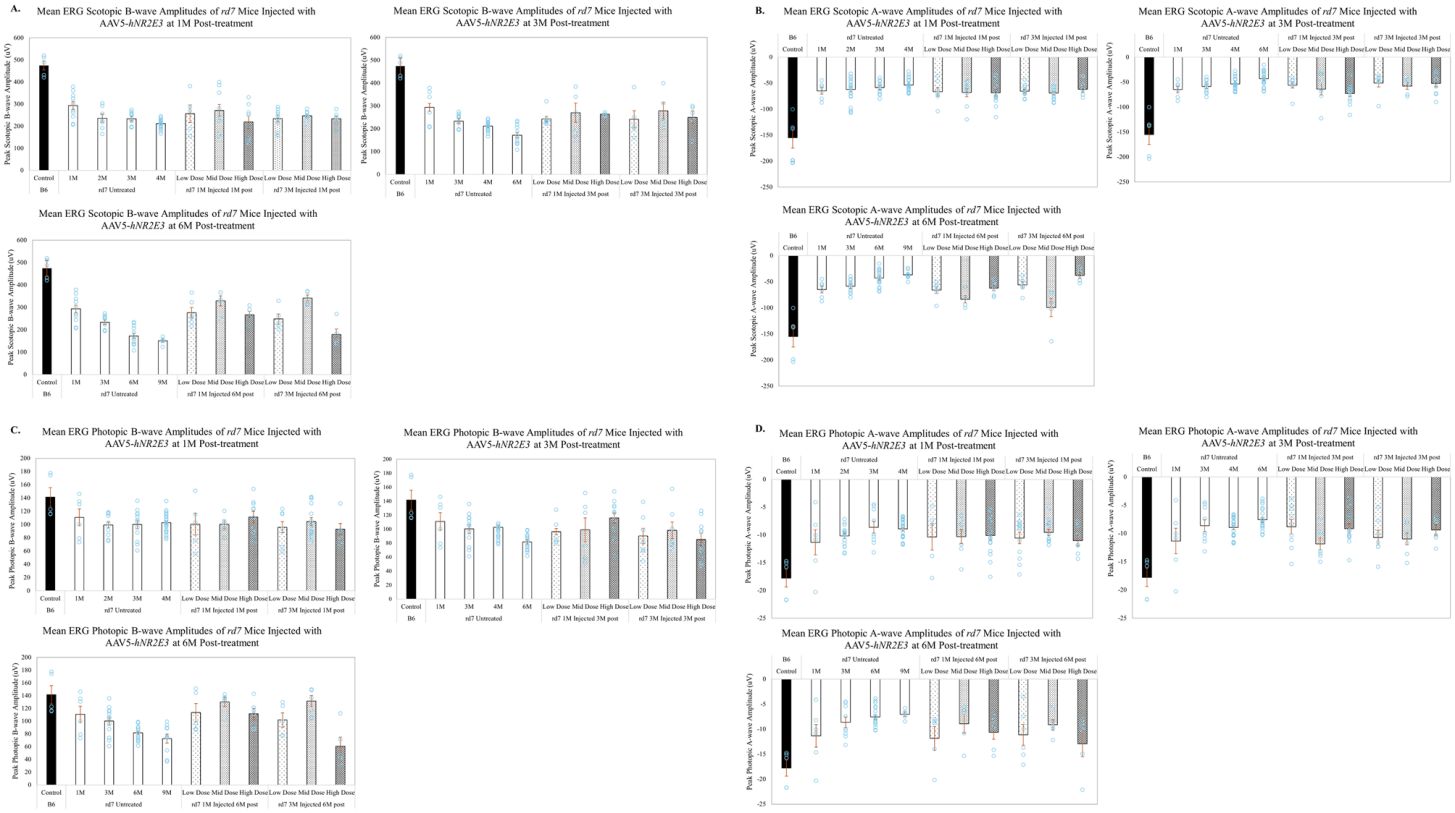
AAV5-*hNR2E3* improved peak scotopic b-wave amplitude in *rd7* mice for all doses. C57BL6/J (B6) animals were used for wild-type controls. (**A**) The scotopic b-wave amplitudes of *rd7* animals injected at P30 were assessed 1-, 3-, and 6-months post injection for the low, mid, and high dose (animal ages 2, 4, and 7 months). *rd7* animals injected at 3-months of age were similarly assessed 1-, 3-, and 6-months post injection for the low, mid, and high dose (animal ages 4, 6, and 9 months). Treated eyes showed significant improvement of retinal function at the 6-month post-injection time points for both the 1 month-injected low and mid dose groups and 3 month-injected mid dose group when compared with age-matched untreated eyes. Intermediate AAV5-*hNR2E3* treated 3 months post *rd7* mice untreated vs mid dose p < 0.05. Early AAV5-*hNR2E3* treated 6 months post *rd7* mice untreated vs low dose p < 0.003, untreated vs mid dose p < 0.0001 and untreated vs high dose p < 0.02. Intermediate AAV5-*hNR2E3* treated 6 months post *rd7* mice untreated vs low dose p < 0.03 and untreated vs mid dose p < 0.0001; low vs mid dose p < 0.04, and mid vs high dose p < 0.0002. Dots represent individual data points for each experimental group. (**B**) The scotopic a-wave amplitudes of *rd7* mice receiving early or intermediate *NR2E3* treatment. Early *NR2E3* treated 6M post *rd7* mice untreated vs mid dose p < 0.009. Intermediate *NR2E3* treated 6M post *rd7* mice untreated vs mid dose p < 0.003 and mid dose vs high dose p < 0.02. (**C**) The photopic b-wave amplitudes of early and intermediate *NR2E3*-treated *rd7* mice assessed at 1, 3 and 6 months post-treatment. Early *NR2E3* treated 6M post *rd7* mice untreated vs mid dose p < 0.002. Intermediate *NR2E3* treated 6M post *rd7* mice untreated vs mid dose p < 0.007 and mid dose vs high dose p < 0.005. (**D**) The photopic a-wave amplitude of early and intermediate *NR2E3* treated *rd7* mice at 1, 3 and 6 months post-treatment. Low Dose = 1 × 10^8^ v/gc; Mid Dose = 1 × 10^9^ v/gc, High Dose = 4 × 10^9^ v/gc. Results are mean ± SEM. n ≥ 5.

The *rd7* mice exhibit progressive loss of rod and cone function as measured by abnormal ERG responses with significant loss of function between 6–12 months of age^18,50,55^. Our previous studies of *rd7* mice treated with *Nr2e3* showed improved ERG responses measured up to 3-months post treatment^16,18^. In this study, dark-adapted and light-adapted ERGs were used to assess photoreceptor function of treated *rd7* retinas by examining rod- and cone-driven responses up to 6-months post treatment (Fig. 8A–D; Supplemental Fig. S3). Similarly to cell counts, *rd7* animals treated during early stage (P30) progression demonstrated statistically significant rescue of dark-adapted photoreceptor function in low, mid and high dose treated retinas (p < 0.0001 for all doses) compared to untreated 6 months post treatment (Fig. 8A). *rd7* mice treated at intermediate stage (3-months of age) of disease only showed significant rescue 6-months post treatment for the low and mid dose groups compared to untreated retinas (Fig. 8A, p < 0.0001). A significant improvement in the dark-adapted a-wave function and light-adapted b-wave function was also observed in early and intermediate mid dose treated *rd7* retinas compared with untreated retinas (Fig. 8B,C). Significant improvement in photoreceptor function was often not detectable until 6-months post treatment for both administration time points; this could be attributed to the delay in expression of AAV5-*hNR2E3*^42,56^. The appearance of improved function and lack of significance for some of the doses and time points could be affected by technical aspects of delivery and measuring function via ERG. The focal delivery of *NR2E3* via subretinal injection will only rescue a localized portion of the retina whereas full-field ERG measures overall photoreceptor function across the entire retina. The different levels of rescue observed in ERGs for each of the doses and time points is the result of the varying amounts of rescue achieved by localized delivery. Interestingly, animals injected at P30 showed increased improvement in ERG response over time for all doses, perhaps supporting the idea that earlier therapeutic intervention has a chance of improving clinical outcome.

## Discussion

The outcomes of inherited diseases such as RP are subject to the influence of several factors including allelic heterogeneity, environment, epigenetic factors, and gene mutations^57–60^. *Nr2e3* is a powerful nuclear hormone receptor that modulates several key gene networks important for maintaining homeostasis in the retina including apoptosis, cell survival, ER stress, immunity, metabolism, neuroprotection, oxidative stress, and phototransduction^16,61^. Our recent study demonstrated that administering *Nr2e3* gene therapy prior to disease onset or during early disease rescues degeneration in the *rd7* mouse model which has an *Nr2e3*-associated gene mutation^16^. This current study was a pharmacological preclinical study that informs dose selection and longitudinal effectiveness of *NR2E3* therapeutic. The novel focus of this study was to examine the efficacy and longitudinal effects of dosage and early versus intermediate therapeutic intervention. All doses showed improvement of photoreceptor survival, and retinal function as well as partial restoration of retinal structure. Furthermore, no gross abnormalities were observed with any of the doses. Sustained improvement was observed up to 6 months after treatment in early and intermediate disease stage groups. It is important to note that focal delivery of *NR2E3* via sub-retinal injection does not rescue the entire retina and manifests as regions of rescue localized around the injection site. This is demonstrated in the functional, histological, and molecular analysis. Localized rescue of photoreceptors illustrate why statistically significant improvements in retinal morphology were observed compared to more limited significance in full field ERGs that measures function across the entire retina. ERGs are also very technically sensitive, and variation demonstrated by the error bars is likely a result of different personnel performing the experiments. Additionally, that it can take AAVs up to 2–4 weeks to begin showing expression likely contributes to the observations of statistically significant improvements after 6 months irrespective of disease stage at time of administration^42,56^.

The *rd7* mouse is a functional null representing varied phenotypes of recessive *NR2E3*-associated retinal disease that encompasses both RP and ESCS^18,19,23,26,50^. The *rd7* mouse is unique in that it has two distinct phenotypes: a developmental over-proliferation of blue cones followed by a slow progressive degeneration of all photoreceptors. It is important to note, *rd7* mice, unlike many other RP models, actually start with relatively normal expression of rhodopsin that degenerate over time along with cones. It has been suggested that *rd7* mice possess some hybrid photoreceptor cells that co-express blue opsin and rhodopsin^62,63^; however, our prior studies could not confirm these findings nor did we observe any similarities between our individually labeled IHCs from this study (Supplemental Fig. S4)^17,64^. Regardless, *rd7* mice have a developmental problem of over-proliferation of blue opsin expressing cone cells due to lack of *Nr2e3* in retinal progenitors^17^. Once matured, *rd7* photoreceptor cells degenerate, and that is likely due to disruption of *Nr2e3* function in many pathways associated with normal function of rod and cone photoreceptor cells. The mechanism of disease in *rd7* thus involves the lack of functional *Nr2e3* protein that disrupts progenitor cell proliferation, rod and cone differentiation, and rod and cone function and survival^17,18^.

Our work and the work of others demonstrates that *NR2E3* plays a role in both the developing and adult retina as well as functions as either an activator or repressor in different photoreceptor development stages^17,64,65^. *Nr2e3* targets genes involved in key homeostasis pathways like cell survival, apoptosis, phototransduction, immunity, neuroprotection, ER stress, oxidative stress, metabolism, and immunity which could be a contributor to the degeneration of rods and cones following blue cone over-proliferation in *rd7* mice when *Nr2e3* proteins are nonfunctional^16^. Importantly, our recent studies show that many RP models have little to no expression of key retinal transcription factors (including *Nr2e3. CRX, Nrl, Rora, Nr1d1*) and are reset by *NR2E3* therapy^16^. Taking the *rd7* disease phenotypes into consideration, and as we treated post photoreceptor development (P30 or 3 months). *NR2E3,* like all NHRs, regulates multiple genes in pathways impacting cell homeostasis^66–68^, as demonstrated in our prior study^16^. In the current study, the mechanism of *NR2E3* rescue of retinal degeneration in *rd7* is likely occurring similarly through modulation of the *Nr2e3* regulated genes and gene networks to a more homeostatic state that attenuates degeneration^16,17,23,64^.

As expected, earlier treatment of *rd7* mice with *NR2E3* is more effective at alleviating or ameliorating disease progression. Treatment during early disease state showed better sustained rescue over time and was able to return ONL thickness and ERG responses to normal levels. It is imperative to note that *NR2E3* is a promising treatment for early or intermediate stage degeneration meaning that it can be used to treat early-stage diagnosis patients and patients that have already been diagnosed and progressed to the later stages of degeneration, which is uncommon for many therapeutics. *NR2E3* was also able to provide rescue for at least 6 months in both progression stage treatments and rescued photoreceptor survival and function to near normal levels in the intermediate stage.

In conclusion, this study demonstrates that AAV5-*hNR2E3* in various doses can treat early and intermediate stage retinal degeneration in the *rd7* mouse for at least 6 months. The results reveal that *NR2E3* gene therapy in the retina can halt degeneration and improve photoreceptor survival and function. Future studies will evaluate whether *NR2E3* reverses degeneration and efficacy of combination therapies. Further studies will also investigate the effectiveness of *NR2E3* therapeutic in other non-*NR2E3*-associated retinal diseases. The results of this pharmacology study are translational to dosage selection of *NR2E3* as a therapeutic agent, longitudinal therapeutic effectiveness, and treatment effectiveness in early and intermediate stage *NR2E3*-associated retinal disease in clinical trials. Following this study, clinical trials of AAV5-*hNR2E3* were initiated and are currently underway to examine the safety and efficacy of the therapeutic for treating various forms of Retinitis Pigmentosa and Leber Congenital Amaurosis (ClinicalTrials.gov Identifier: NCT05203939).

## Materials and methods

### Animal maintenance

This study was carried out in strict accordance with the recommendations in the Guide for the Care and Use of Laboratory Animals of the National Institute of Health, as well as the Association for Research in Vision and Ophthalmology (ARVO) Statement for the Use of Animals in Ophthalmic and Vision Research. All authors complied with the guidelines for Animal Research: Reporting of In Vivo Experiments (ARRIVE). Animals were housed and bred under standard conditions, temperatures within 68–74°F and 12-h light/12-h dark cycles, in the Schepens Eye Research Institute vivarium for the duration of this study. The Schepens Eye Research Institute Animal Care and Use Committee (Protocol Number: 2020N000178) approved animal use and procedures for this study in compliance with the Animal Welfare Act Regulations. C57BL6/J (B6; Jax stock #000664), and *Nr2e3*^*rd7*^/J (*rd7*; Jax stock #002139) mice were ordered from Jackson Laboratories, Bar Harbor, ME.

### Scientific rigor and reproducibility

G*Power software analysis was used to conduct a power calculation for estimating the required sample size for each analysis described (G*Power, version 3.1, https://www.psychologie.hhu.de/arbeitsgruppen/allgemeine-psychologie-und-arbeitspsychologie/gpower). Based on means and standard deviation previously defined in published studies, a minimum of 4 animals were used per experimental group to provide 90% power and 30% difference at a significance level of 0.05. Every procedure was performed using a standardized protocol. This study was performed by several trained individuals in a double blinded and randomized manner to prevent any bias. At least 4–5 biological replicates were studied for each dose and time point to achieve statistical significance. Animals with cataracts, unresolved surgical trauma, or that died prematurely were excluded from the study. Based on these criteria, approximately 20% of the 120 treated animals in this study were excluded from the final analysis. Males (~ 47.37%) and females (~ 52.63%) were used equally, and a gender bias was not observed.

### Statistical analysis

Graphs were analyzed for statistical significance using a two way analysis of variance (ANOVA) (GraphPad Prism, version 9.0, https://www.graphpad.com/). Comparisons were made between the mean peak scotopic or photopic a- or b-wave amplitudes of untreated and treated animals for each treatment and collection time point. Analysis of the differences between the 6-month post time point histological and opsin-positive cell counts, and mean fluorescence intensity of opsins was also performed for each dose compared to the untreated group (Supplemental Tables S1–S10).

### Genotyping

Mouse tail biopsy samples were used for DNA isolation using a quick lysis sodium hydroxide method. Isolated DNA samples were amplified using the primers nr4F: GTAGCCTCTCCTGCTCTGGCAG and *rd7*del4R: CAGGTTGGAAAACACAGGCAAG^18^. For PCR amplification approximately 35 ng of DNA was used in a 10 μl reaction volume containing 10× buffer with MgCl_2_, 40 mM dNTP mix, 10 μM each of forward and reverse primer*,* and 5 U/ml AmpliTaq DNA polymerase. Reactions were denatured at 95 °C for 3 min followed by 30 cycles at 95 °C for 30 s, 60 °C for 30 s, 72 °C for 42 s and a final extension at 72 °C for 5 min. Amplicons were separated using a 2% agarose gel and visualized under UV light after staining with ethidium bromide. The wild-type amplicon is 339 base pairs (bp) in size and the mutant amplicon is 239 bp. As the *rd7* mutation is a 9 kb line element insertion in the region amplified, it will not produce an *Nr2e3* PCR amplicon under these conditions, and the amplicon from *rd7* mice is a pseudogene^18,49^.

### AAV5-*hNR2E3* dose preparation

The drug product consists of adeno associated virus 5 (AAV5) vector containing the human *NR2E3* gene (AAV5-*hNR2E3* driven by the CAG promoter). The GLP vector drug product was provided by Ocugen, INC. in three different concentrations (2 × 10^11^ Vg/ml, 2 × 10^12^ Vg/ml, 8 × 10^12^ Vg/ml) diluted with formulation buffer (sterile solution containing 10 mM sodium phosphate, 180 mM sodium chloride, and 0.001% Poloxamer 188 (PF-68) at pH 7.3 ± 0.5). Upon arrival, the three concentrations were diluted into a low (1 × 10^8^ vg/eye), medium (1 × 10^9^ vg/eye), and high (4 × 10^9^ vg/eye) dose.

### Subretinal injection

AAV5-*hNR2E3* was delivered by subretinal injection into the right eye of *rd7* mice. Mice were injected at either P30 to treat early-stage disease progression or 3-months of age to treat intermediate progression. Control injections consisted of no injection in the contralateral (left) eye, and mock injection in the contralateral eye with saline buffer (Supplemental Fig. S5). Adults aged to P30 or 3 months old were anesthetized by intraperitoneal (IP) injection using a ketamine/xylazine mixture. A sterile 30G needle was used to make an incision in the sclera just posterior to the limbus, and a Hamilton syringe with a blunt 33G cannula attached was inserted into the incision^16^. A total volume of 0.5 µl of therapeutic product was manually injected into the subretinal space of the adult mice for each dosage group: low (1 × 10^8^ vg/eye), medium (1 × 10^9^ vg/eye), and high (4 × 10^9^ vg/eye) dose. Accessing the central retinal region with an injection in the mouse is difficult. Therefore, the location of rescue often appeared between the peripheral and central retina, sometimes extended into the central retina based on the degree of rescue achieved by the focal injection.

### Clinical examination

All animals (treated, untreated, wildtype (WT) B6 controls) underwent fundus examination and optical coherence tomography (OCT). Animals were anesthetized with a ketamine/xylazine mixture by IP injection and 1% tropicamide was used to dilate pupils. The Micron III Retinal Imaging Camera (Phoenix Research Laboratories, Pleasanton, CA, USA) and Stream Pix software (version 5, https://www.norpix.com/products/streampix/streampix.php) were used for fundus imaging. The Bioptigen OCT scanner (Bioptigen Inc, Durham, North Carolina, USA) and software (Envisu Invivovue, version 2, https://www.leica-microsystems.com/products/surgical-microscopes/p/envisu-r-class/) were used to perform OCT. A mounting tube with a bite bar restrained mice while the real time OCT image was used for alignment of the retina. Each eye had four rotational cross section scans (nasal-caudal and dorsal–ventral) taken with 100 series/scan^18^. The Bioptigen OCT software analyzed the retinal scans and representative images were taken of the central retina near the optic nerve.

### Electroretinography

Mice were examined through electroretinography analysis as previously described^16,17^. Animals were dark-adapted overnight or for a minimum of 4 h before being anesthetized. Anesthesia was administered through an IP injection of a mixture of ketamine and xylazine, and pupils were dilated with 1% tropicamide. Genteal was used as a lubricant to prevent cataracts forming during the procedure. The reference electrode was placed subcutaneously in the forehead and the ground electrode was inserted subcutaneously in the tail base. Gold loop electrodes (Diagnosys LLC) were placed on the corneal apex. The Espion Visual Electrophysiology System (Diagnosys LLC, Lowell, MA, USA) performed dark- and light-adapted ERGs. Dark- and light-adapted responses were recorded according to the same protocol used previously^16^. Briefly, responses were recorded at intensities between 0.000249 and 24.1 cd s/m^2^ in 4.0 log intensity increments for dark-adapted. Using the same 4.0 log intensity difference, light-adapted responses were obtained between 0.1 and 25.6 cd s/m^2^ following 7 min of adaptation to background light. The peak a- and b-wave amplitude from each treated animal was selected between intensities of 0.061 cd s/m^2^ and 24.1 cd s/m^2^ for scotopic responses and 0.1 cd s/m^2^ and 25.6 cd s/m^2^ for photopic responses, averaged for each dose and time point, and plotted in comparison to peak amplitudes from wild type and untreated *rd7* mice.

### Histology

Histological analysis was performed as previously described^16^. Directly following euthanasia, a cautery mark was made on the eyes for dorsal orientation and then eyes were enucleated. Tissues from AAV5-*hNR2E3* treated animals were immediately immersed in 4% paraformaldehyde (PFA) diluted with 1× phosphate buffered solution (PBS) and left overnight at 4 °C. Untreated tissue was immersed in 4% PFA and similarly left overnight at 4 °C. Eyes were paraffin embedded the following day with dorsal/ventral orientation using the cautery mark made after euthanasia. Embedded eyes were trimmed in the sagittal plane to a depth of 100 µm. 5 µm serial sections were collected at about 100 µm of retinal depth. Deparaffination of retina sections was performed in xylene and ethanol washes followed by staining with hematoxylin and eosin Y. Stained sections were mounted with Permount mounting medium^16^. Visualization of the rescued regions and image capture of the slides was performed with the Leica DMI6000 microscope (Leica Microsystems, Wetzlar, Germany). Identification of the rescued regions was performed visually prior to image captures to ensure that images were of the rescued regions. Cell counts were accomplished in a double-blinded manner with at minimum three individuals. Cell layer number in the outer nuclear layer (ONL) was counted for treated and untreated samples and B6 control samples. In untreated samples, cell layer counts were done for non-whorl regions of the retina. Percent observed rescue was quantified by comparing ONL cell layer numbers in treated samples to untreated and B6 control ONL counts.

### Immunohistochemistry

Immunohistochemistry (IHC) analysis was performed on 5 µm retinal serial sections from the same tissue samples used for histology staining previously described. Tissue sections were blocked for 1 h with 2% normal horse serum (S-2000 Vector Labs, CA) in 1× PBS then, incubated overnight in a 1:200 dilution of the following cell type-specific primary antibodies: blue opsin (rabbit polyclonal, Millipore AB5407); green/red opsin (rabbit polyclonal, Millipore AB5405); and rhodopsin (mouse monoclonal, Millipore MAB5356). On the second day, sections were rinsed with 1X PBS and incubated for 1 h in the dark in a 1:400 dilution of the following corresponding secondary antibodies: mouse 488 (Alexa Fluor 488 goat anti-mouse, Invitrogen A11001); and rabbit 488 (Alexa Fluor 488 goat anti-rabbit, Invitrogen A11008). Sections were rinsed with PBS and nuclei labeled with 4,6-Diamidino-2-Phenylindole, Dihydrochloride (DAPI, dilactate, Invitrogen D3571). Antibody labeling was visualized, and representative images captured using a Leica DMI6000 fluorescent microscope (Leica Microsystems, Wetzlar, Germany) or a Nikon i90 microscope (Melville, New York, USA) equipped with fluorochrome appropriate band-pass filters^16^. Analysis of IHC images at approximately 100 µm retinal depth was performed by 2–3 individuals in a double-blinded manner. Fluorescence intensity of the opsins was measured using ImageJ to analyze mean gray values in each dose for both early and intermediate treatment groups at the 6 months post time point compared to untreated controls. The mean gray levels were between 0 and 85, with 0 indicating absence of fluorescence and 85 indicating maximum fluorescence intensity. The number of blue and green opsin-positive cells was also counted for the early and intermediate treatment groups at 6 months post-treatment.

### Ethical approval

This study was carried out in strict accordance with the recommendations in the Guide for the Care and Use of Laboratory Animals of the National Institute of Health, as well as the Association for Research in Vision and Ophthalmology (ARVO) Statement for the Use of Animals in Ophthalmic and Vision Research.

### Supplementary Information

Supplementary Figure S1.

Supplementary Figure S2.

Supplementary Figure S3.

Supplementary Figure S4.

Supplementary Figure S5.

Supplementary Legends.

Supplementary Tables.

## Data Availability

All data used in this paper has been presented in the figures and supplementary files.
