# Supplementary figures and images for: Evaluating therapeutic potential of NR2E3 doses in the rd7 mouse model of retinal degeneration
Source: Sci Rep. 2024 Jul 17;14:16490. doi: 10.1038/s41598-024-67095-6 (PMC11254931; doi:10.1038/s41598-024-67095-6)

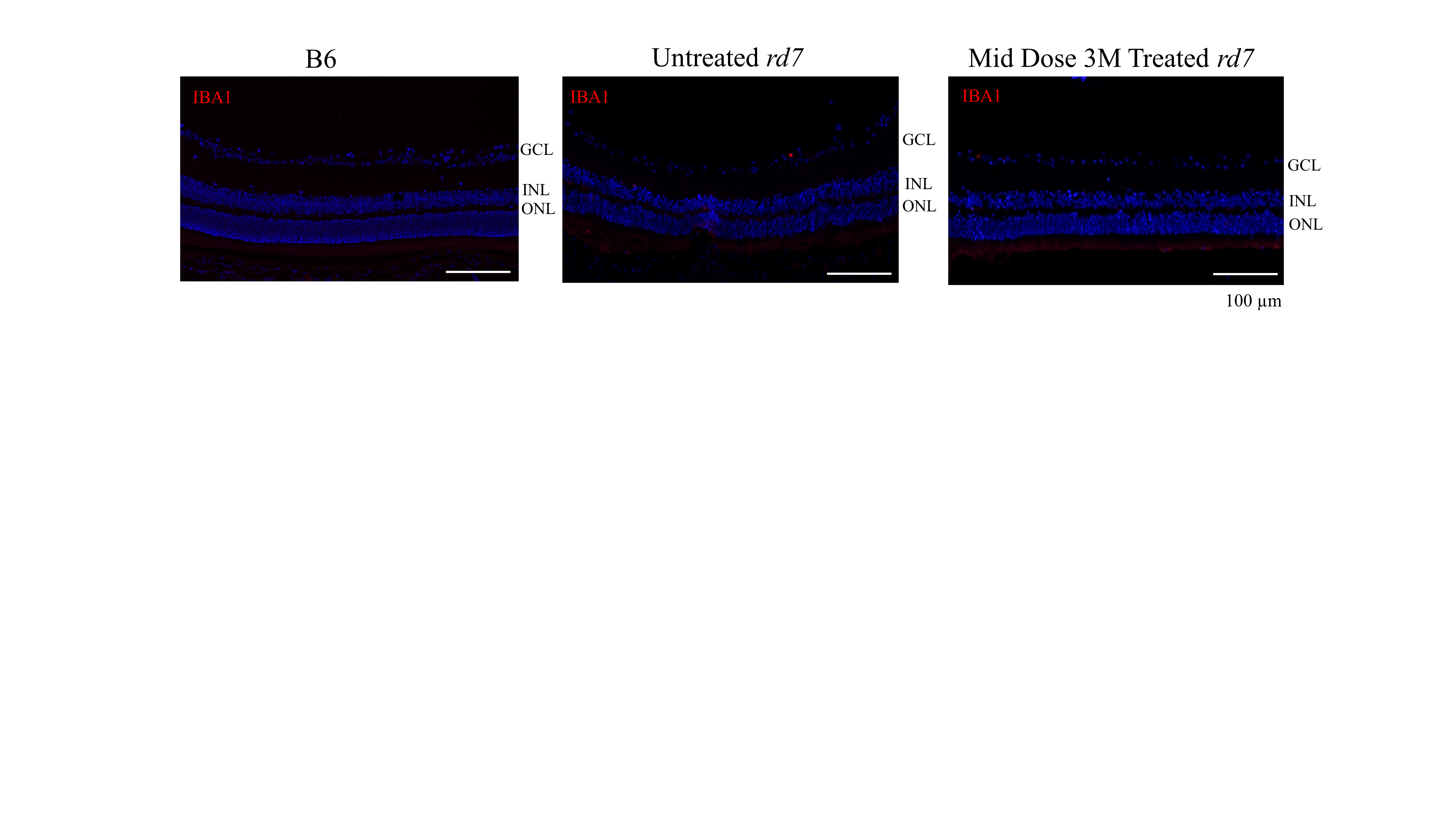

Supplement: Supplementary file 1 — Supplementary Figure S1. [file 41598_2024_67095_MOESM1_ESM.tif]

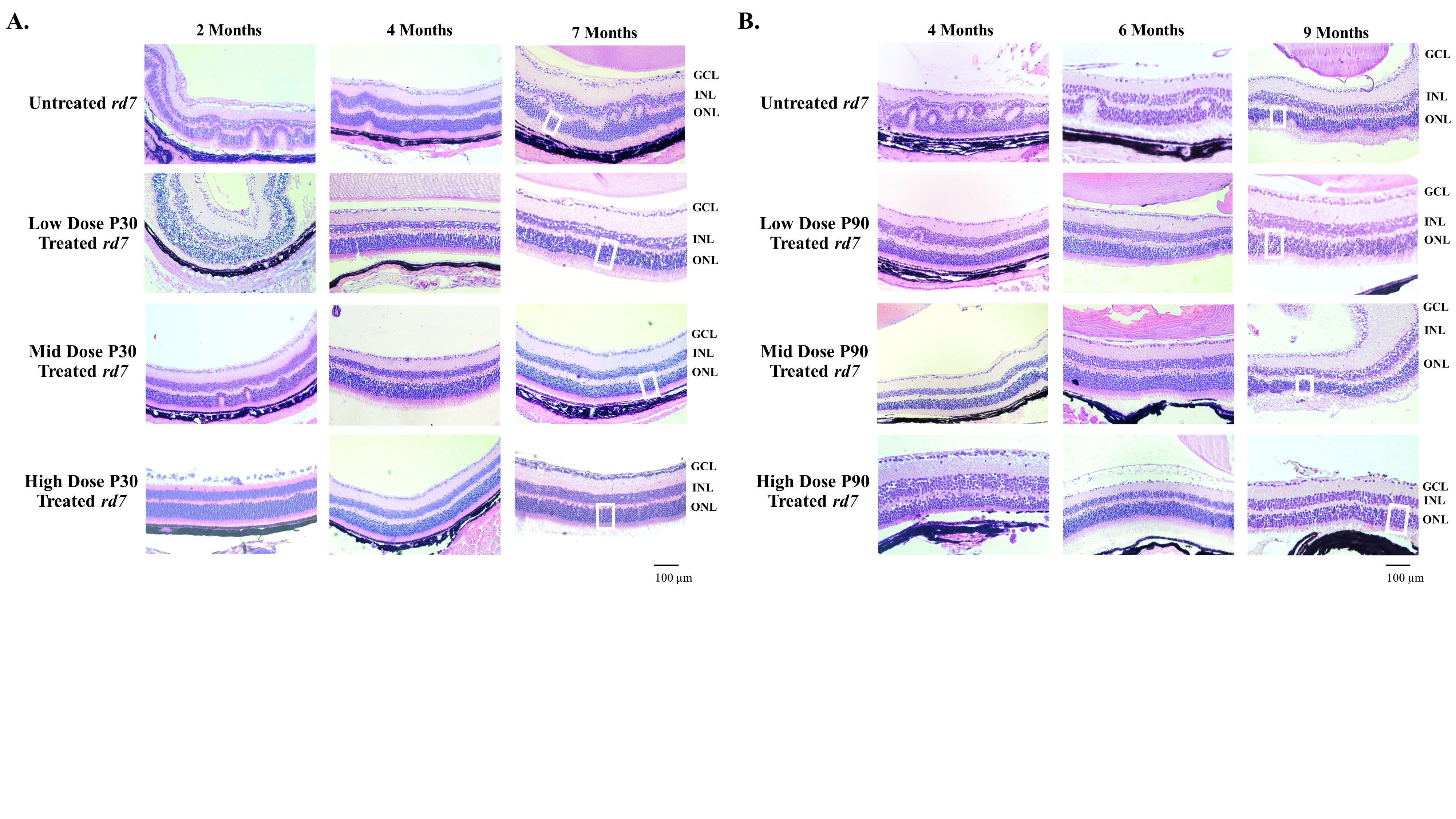

Supplement: Supplementary file 2 — Supplementary Figure S2. [file 41598_2024_67095_MOESM2_ESM.tif]

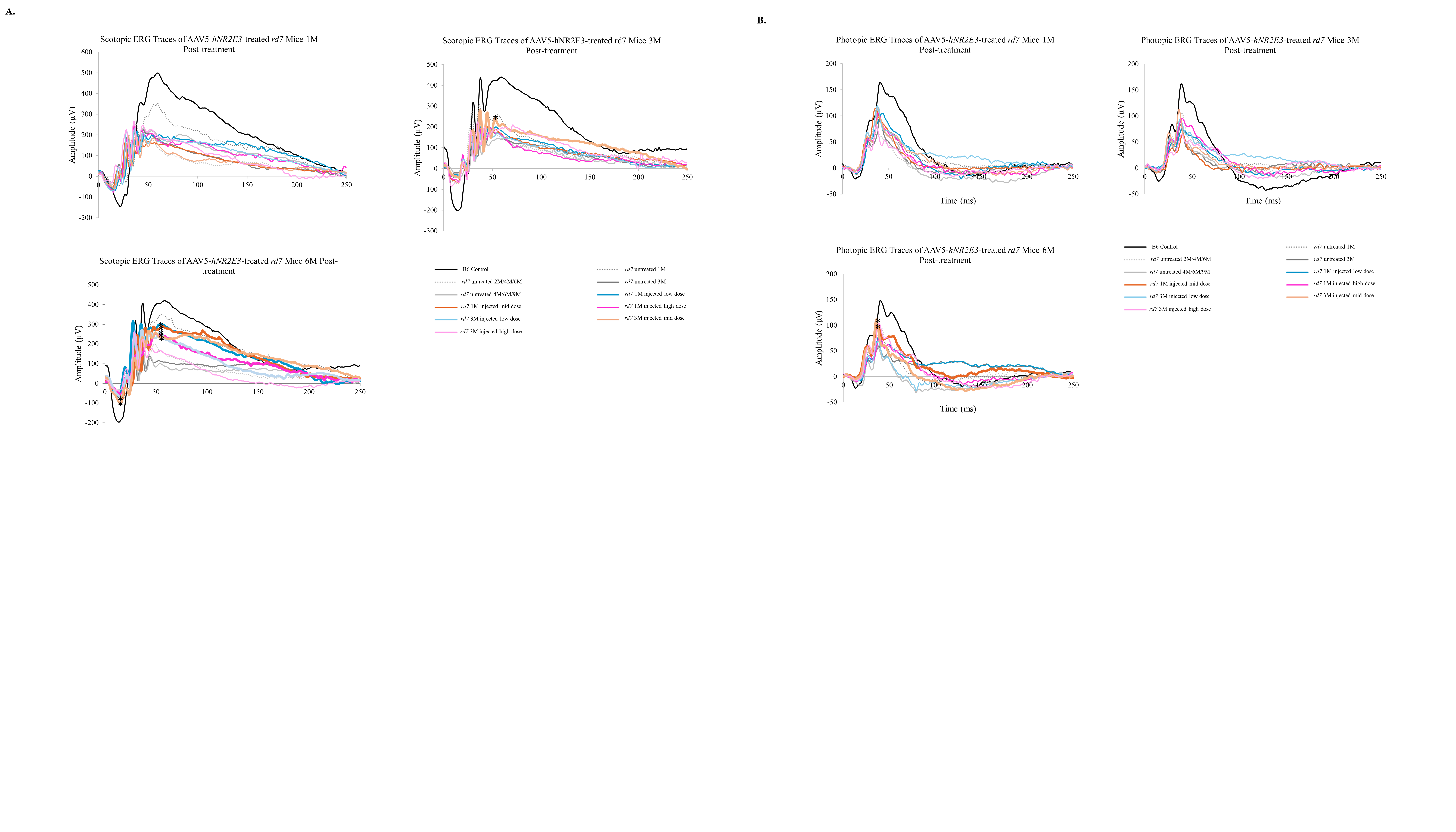

Supplement: Supplementary file 3 — Supplementary Figure S3. [file 41598_2024_67095_MOESM3_ESM.tif]

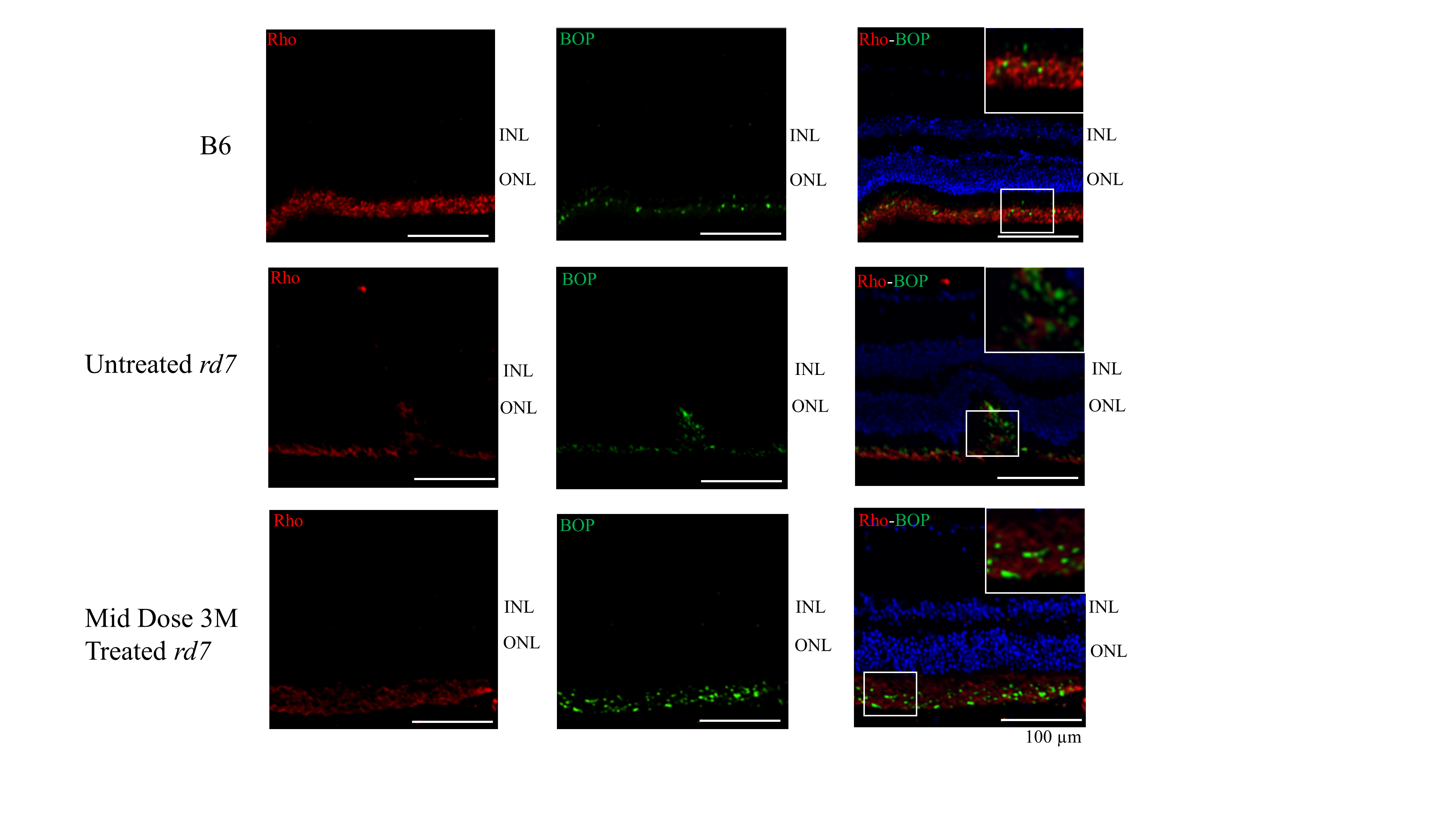

Supplement: Supplementary file 4 — Supplementary Figure S4. [file 41598_2024_67095_MOESM4_ESM.tif]

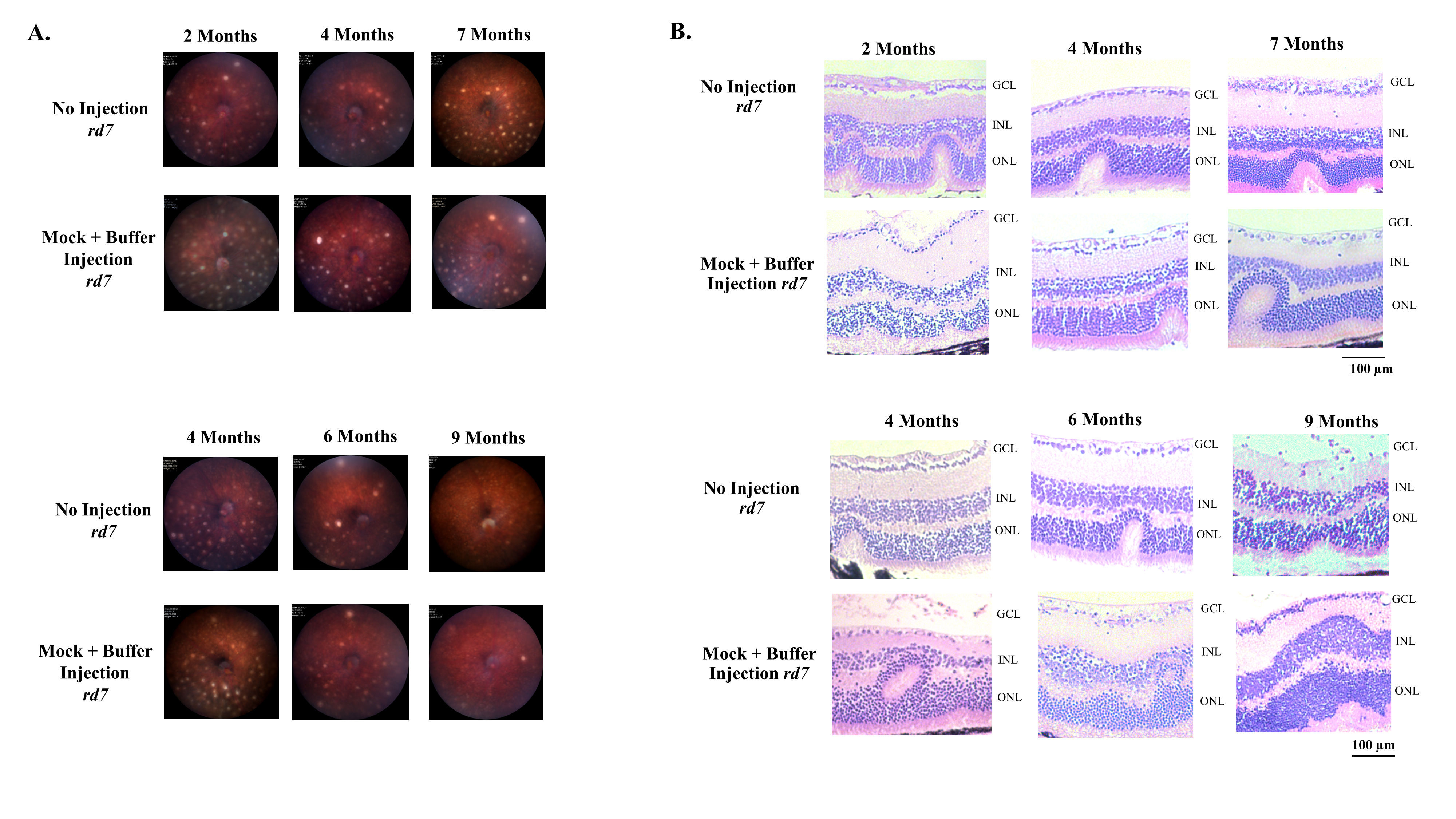

Supplement: Supplementary file 5 — Supplementary Figure S5. [file 41598_2024_67095_MOESM5_ESM.tif]
