# Supplementary material for: Evaluating therapeutic potential of NR2E3 doses in the rd7 mouse model of retinal degeneration
Source: Sci Rep. 2024 Jul 17;14:16490. doi: 10.1038/s41598-024-67095-6 (PMC11254931; doi:10.1038/s41598-024-67095-6)
Supplement: Supplementary file 6 — Supplementary Legends. [file 41598_2024_67095_MOESM6_ESM.docx]

**Supplemental Figure Legends**

**Supplemental Figure S1. No change in expression of the inflammatory marker *IBA1* in AAV5-*hNR2E3*-treated *rd7* retinas.** Immunohistochemistry (IHC) for IBA1 shows no evidence of inflammation in intermediate *NR2E3* mid dose treated *rd7* retinas assessed at 6 months post treatment. GCL: Ganglion Cell Layer; INL: Inner Nuclear Layer; ONL: Outer Nuclear Layer. Scale bars 100 µm. n≥4.

**Supplemental Figure S2. Lower magnification of AAV5-*hNR2E3* treatment at P30 or P90 showing reduced abnormal retinal morphology in *rd7* treated retinas in low, mid, and high doses.** A. H/E of *rd7* animals injected at P30 and collected 1-, 3-, and 6-months post injection (animal ages 2, 4, and 7 months) with low, mid, or high dose therapy, and age matched untreated *rd7* controls. B. *rd7* animals injected at 3-months of age (P90) and collected 1-, 3-, and 6-months post injection (animal ages 4, 6, and 9 months) with low, mid, or high dose therapy, and age matched untreated *rd7* controls. White boxes in the 7- and 9-month time point images represent the respective locations of ONL counts. Low Dose = 1 × 10^8^ v/gc; Mid Dose = 1 × 10^9^ v/gc, High Dose = 4 × 10^9^ v/gc. GCL: Ganglion Cell Layer; INL: Inner Nuclear Layer; ONL: Outer Nuclear Layer. Scale bar = 100µm. n≥5.

**Figure S3. Representative ERG responses in AAV5-*hNR2E3*-treated *rd7* retinas.** A) Representative scotopic ERG response traces of early and intermediate AAV5-*hNR2E3*-treated *rd7* retinas at 1, 3 and 6 months post-treatment. B) Representative photopic ERG response traces of early and intermediate AAV5-*hNR2E3*-treated *rd7* retinas at 1, 3 and 6 months post-treatment. The statistically significant groups are indicated with a thicker line and black asterisks.

**Supplemental Figure S4. Absence of overlapping rhodopsin and blue opsin expression in untreated and *NR2E3*-treated *rd7* retinas.** IHC co-labeling for rhodopsin and blue opsin shows no overlap in expression in both untreated and intermediate *NR2E3* mid dose-treated *rd7* retinas assessed at 1 month post treatment. INL: Inner Nuclear Layer; ONL: Outer Nuclear Layer. Scale bars 100 µm. n≥4.

**Supplemental Figure S5. No observable difference between no injection and mock injection with buffer in *rd7* untreated eyes.** A. No major differences were observed in the fundus of untreated *rd7* eyes receiving no injection or a mock injection with buffer at 2-, 4- and 7-months post treatment, as well as 4-, 6- and 9-months post treatment. B. H&E staining did not show any observable differences between untreated *rd7* eyes receiving no injection or a mock injection with buffer at 2-, 4- and 7-months post treatment, as well as 4-, 6- and 9-months post treatment. GCL: Ganglion Cell Layer; INL: Inner Nuclear Layer; ONL: Outer Nuclear Layer. Scale bar = 100 µm. n≥5.
