# Supplementary material for: Evaluating therapeutic potential of NR2E3 doses in the rd7 mouse model of retinal degeneration
Source: Sci Rep. 2024 Jul 17;14:16490. doi: 10.1038/s41598-024-67095-6 (PMC11254931; doi:10.1038/s41598-024-67095-6)
Supplement: Supplementary file 7 — Supplementary Tables. [file 41598_2024_67095_MOESM7_ESM.docx]

**Table S1. Two-way analysis of variance (ANOVA) post-hoc test results for 6M post *NR2E3* treated *rd7* histological cell counts.**

| **Treatment time** | **Tukey's multiple comparisons test** | **Mean Diff.** | **95.00% CI of diff.** | **Adjusted P Value** |
| --- | --- | --- | --- | --- |
| 1M | B6 vs *rd7* untreated | 4.887 | 1.683 to 8.091 | 0.0011 |
|  | B6 vs *rd7* Low Dose | 0.4200 | -3.129 to 3.969 | 0.9999 |
|  | B6 vs *rd7* Mid Dose | -1.700 | -5.046 to 1.646 | 0.6798 |
|  | B6 vs *rd7* High Dose | 0.8867 | -2.460 to 4.233 | 0.9833 |
|  | *Rd7* Untreated vs. Low Dose | -4.467 | -7.882 to -1.051 | 0.0054 |
|  | *Rd7* Untreated vs. Mid Dose | -6.587 | -9.791 to -3.383 | <0.0001 |
|  | *Rd7* Untreated vs. High Dose | -4.000 | -7.204 to -0.7961 | 0.0084 |
|  | *Rd7* Low Dose vs. Mid Dose | -2.120 | -5.669 to 1.429 | 0.4976 |
|  | *Rd7* Low Dose vs. High Dose | 0.4667 | -3.083 to 4.016 | 0.9998 |
|  | *Rd7* Mid Dose vs. High Dose | 2.587 | -0.7597 to 5.933 | 0.2107 |
|  |  |  |  |  |
| 3M | B6 vs *rd7* untreated | 5.245 | 2.841 to 7.649 | <0.0001 |
|  | B6 vs *rd7* Low Dose | -0.4733 | -3.141 to 2.194 | 0.9987 |
|  | B6 vs *rd7* Mid Dose | 2.740 | 0.07255 to 5.407 | 0.0412 |
|  | B6 vs *rd7* High Dose | 3.527 | 0.8592 to 6.194 | 0.0044 |
|  | *Rd7* Untreated vs. Low Dose | -5.718 | -8.123 to -3.314 | <0.0001 |
|  | *Rd7* Untreated vs. Mid Dose | -2.505 | -4.909 to -0.1006 | 0.0370 |
|  | *Rd7* Untreated vs. High Dose | -1.718 | -4.123 to 0.6861 | 0.2983 |
|  | *Rd7* Low Dose vs. Mid Dose | 3.213 | 0.5459 to 5.881 | 0.0109 |
|  | *Rd7* Low Dose vs. High Dose | 4.000 | 1.333 to 6.667 | 0.0011 |
|  | *Rd7* Mid Dose vs. High Dose | 0.7867 | -1.881 to 3.454 | 0.9728 |

**Table S2. ANOVA post-hoc results for 6M post *NR2E3* treated *rd7* blue opsin cell counts.**

| **Treatment time** | **Tukey's multiple comparisons test** | **Mean Diff.** | **95.00% CI of diff.** | **Adjusted P Value** |
| --- | --- | --- | --- | --- |
| 1M | B6 vs *rd7* untreated | 13.67 | 2.367 to 24.97 | 0.0117 |
|  | B6 vs *rd7* Low Dose | 3.400 | -7.900 to 14.70 | 0.9639 |
|  | B6 vs *rd7* Mid Dose | 3.533 | -7.766 to 14.83 | 0.9560 |
|  | B6 vs *rd7* High Dose | 1.067 | -10.23 to 12.37 | >0.9999 |
|  | *Rd7* Untreated vs. Low Dose | -10.27 | -20.05 to -0.4808 | 0.0359 |
|  | *Rd7* Untreated vs. Mid Dose | -10.13 | -19.92 to -0.3475 | 0.0394 |
|  | *Rd7* Untreated vs. High Dose | -12.60 | -22.39 to -2.814 | 0.0067 |
|  | *Rd7* Low Dose vs. Mid Dose | 0.1333 | -9.653 to 9.919 | >0.9999 |
|  | *Rd7* Low Dose vs. High Dose | -2.333 | -12.12 to 7.453 | 0.9900 |
|  | *Rd7* Mid Dose vs. High Dose | -2.467 | -12.25 to 7.319 | 0.9863 |
|  |  |  |  |  |
| 3M | B6 vs *rd7* untreated | 14.13 | 3.733 to 24.53 | 0.0048 |
|  | B6 vs *rd7* Low Dose | 0.8333 | -10.04 to 11.71 | >0.9999 |
|  | B6 vs *rd7* Mid Dose | 4.000 | -6.877 to 14.88 | 0.8912 |
|  | B6 vs *rd7* High Dose | 8.667 | -2.211 to 19.54 | 0.1715 |
|  | *Rd7* Untreated vs. Low Dose | -13.30 | -22.85 to -3.746 | 0.0039 |
|  | *Rd7* Untreated vs. Mid Dose | -10.13 | -19.69 to -0.5796 | 0.0339 |
|  | *Rd7* Untreated vs. High Dose | -5.467 | -15.02 to 4.087 | 0.5131 |
|  | *Rd7* Low Dose vs. Mid Dose | 3.167 | -6.904 to 13.24 | 0.9475 |
|  | *Rd7* Low Dose vs. High Dose | 7.833 | -2.237 to 17.90 | 0.1906 |
|  | *Rd7* Mid Dose vs. High Dose | 4.667 | -5.404 to 14.74 | 0.7336 |

**Table S3. ANOVA post-hoc results for 6M post *NR2E3* treated *rd7* green opsin cell counts.**

| **Treatment time** | **Tukey's multiple comparisons test** | **Mean Diff.** | **95.00% CI of diff.** | **Adjusted P Value** |
| --- | --- | --- | --- | --- |
| 1M | B6 vs *rd7* untreated | 12.27 | 6.902 to 17.63 | <0.0001 |
|  | B6 vs *rd7* Low Dose | 3.417 | -2.238 to 9.071 | 0.4783 |
|  | B6 vs *rd7* Mid Dose | 1.667 | -3.698 to 7.031 | 0.9591 |
|  | B6 vs *rd7* High Dose | 1.944 | -3.217 to 7.106 | 0.8965 |
|  | *Rd7* Untreated vs. Low Dose | -8.850 | -14.21 to -3.486 | 0.0005 |
|  | *Rd7* Untreated vs. Mid Dose | -10.60 | -15.66 to -5.543 | <0.0001 |
|  | *Rd7* Untreated vs. High Dose | -10.32 | -15.16 to -5.480 | <0.0001 |
|  | *Rd7* Low Dose vs. Mid Dose | -1.750 | -7.114 to 3.614 | 0.9476 |
|  | *Rd7* Low Dose vs. High Dose | -1.472 | -6.634 to 3.689 | 0.9740 |
|  | *Rd7* Mid Dose vs. High Dose | 0.2778 | -4.564 to 5.120 | >0.9999 |
|  |  |  |  |  |
| 3M | B6 vs *rd7* untreated | 12.48 | 5.562 to 19.40 | 0.0001 |
|  | B6 vs *rd7* Low Dose | 3.667 | -4.058 to 11.39 | 0.7497 |
|  | B6 vs *rd7* Mid Dose | 2.611 | -6.183 to 11.41 | 0.9700 |
|  | B6 vs *rd7* High Dose | 6.333 | -1.391 to 14.06 | 0.1618 |
|  | *Rd7* Untreated vs. Low Dose | -8.815 | -15.24 to -2.392 | 0.0032 |
|  | *Rd7* Untreated vs. Mid Dose | -9.870 | -17.55 to -2.194 | 0.0062 |
|  | *Rd7* Untreated vs. High Dose | -6.148 | -12.57 to 0.2744 | 0.0670 |
|  | *Rd7* Low Dose vs. Mid Dose | -1.056 | -9.465 to 7.354 | 0.9999 |
|  | *Rd7* Low Dose vs. High Dose | 2.667 | -4.616 to 9.949 | 0.9142 |
|  | *Rd7* Mid Dose vs. High Dose | 3.722 | -4.687 to 12.13 | 0.8069 |

**Table S4. Multiple comparisons for 6M post *NR2E3* treated *rd7* blue opsin mean fluorescence intensity.**

| **Treatment time** | **Tukey's multiple comparisons test** | **Mean Diff.** | **95.00% CI of diff.** | **Adjusted P Value** |
| --- | --- | --- | --- | --- |
| 1M | B6 vs *rd7* untreated | 13.07 | 4.107 to 22.03 | 0.0022 |
|  | B6 vs *rd7* Low Dose | 1.331 | -8.116 to 10.78 | 0.9996 |
|  | B6 vs *rd7* Mid Dose | 1.716 | -7.730 to 11.16 | 0.9980 |
|  | B6 vs *rd7* High Dose | 2.377 | -6.584 to 11.34 | 0.9807 |
|  | *Rd7* Untreated vs. Low Dose | -11.74 | -20.70 to -2.776 | 0.0060 |
|  | *Rd7* Untreated vs. Mid Dose | -11.35 | -20.31 to -2.390 | 0.0081 |
|  | *Rd7* Untreated vs. High Dose | -10.69 | -19.14 to -2.242 | 0.0082 |
|  | *Rd7* Low Dose vs. Mid Dose | 0.3853 | -9.061 to 9.832 | >0.9999 |
|  | *Rd7* Low Dose vs. High Dose | 1.046 | -7.915 to 10.01 | 0.9999 |
|  | *Rd7* Mid Dose vs. High Dose | 0.6611 | -8.301 to 9.623 | >0.9999 |
|  |  |  |  |  |
| 3M | B6 vs *rd7* untreated | 13.55 | 5.348 to 21.76 | 0.0006 |
|  | B6 vs *rd7* Low Dose | 3.854 | -4.351 to 12.06 | 0.7369 |
|  | B6 vs *rd7* Mid Dose | 5.139 | -3.510 to 13.79 | 0.4854 |
|  | B6 vs *rd7* High Dose | 10.65 | 2.006 to 19.30 | 0.0103 |
|  | *Rd7* Untreated vs. Low Dose | -9.699 | -17.43 to -1.963 | 0.0089 |
|  | *Rd7* Untreated vs. Mid Dose | -8.413 | -16.62 to -0.2085 | 0.0423 |
|  | *Rd7* Untreated vs. High Dose | -2.898 | -11.10 to 5.307 | 0.9170 |
|  | *Rd7* Low Dose vs. Mid Dose | 1.285 | -6.920 to 9.490 | 0.9992 |
|  | *Rd7* Low Dose vs. High Dose | 6.800 | -1.404 to 15.01 | 0.1468 |
|  | *Rd7* Mid Dose vs. High Dose | 5.515 | -3.133 to 14.16 | 0.4028 |

**Table S5. Multiple comparisons for 6M post *NR2E3* treated *rd7* green opsin mean fluorescence intensity.**

| **Treatment time** | **Tukey's multiple comparisons test** | **Mean Diff.** | **95.00% CI of diff.** | **Adjusted P Value** |
| --- | --- | --- | --- | --- |
| 1M | B6 vs *rd7* untreated | 12.11 | 1.953 to 22.27 | 0.0133 |
|  | B6 vs *rd7* Low Dose | 1.875 | -9.255 to 13.00 | 0.9988 |
|  | B6 vs *rd7* Mid Dose | 0.9230 | -10.21 to 12.05 | >0.9999 |
|  | B6 vs *rd7* High Dose | -0.6207 | -11.18 to 9.938 | >0.9999 |
|  | *Rd7* Untreated vs. Low Dose | -10.24 | -20.40 to -0.07854 | 0.0475 |
|  | *Rd7* Untreated vs. Mid Dose | -11.19 | -21.35 to -1.030 | 0.0250 |
|  | *Rd7* Untreated vs. High Dose | -12.73 | -22.27 to -3.203 | 0.0048 |
|  | *Rd7* Low Dose vs. Mid Dose | -0.9518 | -12.08 to 10.18 | >0.9999 |
|  | *Rd7* Low Dose vs. High Dose | -2.495 | -13.05 to 8.064 | 0.9905 |
|  | *Rd7* Mid Dose vs. High Dose | -1.544 | -12.10 to 9.015 | 0.9995 |
|  |  |  |  |  |
| 3M | B6 vs *rd7* untreated | 13.11 | 7.412 to 18.80 | <0.0001 |
|  | B6 vs *rd7* Low Dose | 6.032 | -0.5107 to 12.57 | 0.0861 |
|  | B6 vs *rd7* Mid Dose | 9.486 | 2.590 to 16.38 | 0.0028 |
|  | B6 vs *rd7* High Dose | 7.839 | 1.297 to 14.38 | 0.0113 |
|  | *Rd7* Untreated vs. Low Dose | -7.075 | -12.34 to -1.815 | 0.0035 |
|  | *Rd7* Untreated vs. Mid Dose | -3.621 | -9.315 to 2.074 | 0.4391 |
|  | *Rd7* Untreated vs. High Dose | -5.268 | -10.53 to -0.007543 | 0.0495 |
|  | *Rd7* Low Dose vs. Mid Dose | 3.454 | -3.088 to 9.997 | 0.6576 |
|  | *Rd7* Low Dose vs. High Dose | 1.807 | -4.361 to 7.975 | 0.9745 |
|  | *Rd7* Mid Dose vs. High Dose | -1.647 | -8.189 to 4.895 | 0.9892 |

**Table S6. Multiple comparisons for 6M post *NR2E3* treated *rd7* rhodopsin mean fluorescence intensity.**

| **Treatment time** | **Tukey's multiple comparisons test** | **Mean Diff.** | **95.00% CI of diff.** | **Adjusted P Value** |
| --- | --- | --- | --- | --- |
| 1M | B6 vs *rd7* untreated | 10.57 | 2.021 to 19.12 | 0.0086 |
|  | B6 vs *rd7* Low Dose | 0.5376 | -8.769 to 9.845 | >0.9999 |
|  | B6 vs *rd7* Mid Dose | -1.439 | -10.75 to 7.868 | 0.9995 |
|  | B6 vs *rd7* High Dose | 0.4419 | -8.865 to 9.749 | >0.9999 |
|  | *Rd7* Untreated vs. Low Dose | -10.03 | -19.03 to -1.034 | 0.0216 |
|  | *Rd7* Untreated vs. Mid Dose | -12.01 | -21.01 to -3.011 | 0.0040 |
|  | *Rd7* Untreated vs. High Dose | -10.13 | -19.13 to -1.130 | 0.0200 |
|  | *Rd7* Low Dose vs. Mid Dose | -1.977 | -11.70 to 7.744 | 0.9969 |
|  | *Rd7* Low Dose vs. High Dose | -0.09569 | -9.817 to 9.625 | >0.9999 |
|  | *Rd7* Mid Dose vs. High Dose | 1.881 | -7.840 to 11.60 | 0.9977 |
|  |  |  |  |  |
| 3M | B6 vs *rd7* untreated | 12.38 | 3.414 to 21.34 | 0.0030 |
|  | B6 vs *rd7* Low Dose | 1.968 | -6.578 to 10.51 | 0.9929 |
|  | B6 vs *rd7* Mid Dose | 5.065 | -4.491 to 14.62 | 0.6404 |
|  | B6 vs *rd7* High Dose | 2.427 | -6.537 to 11.39 | 0.9819 |
|  | *Rd7* Untreated vs. Low Dose | -10.41 | -19.37 to -1.446 | 0.0157 |
|  | *Rd7* Untreated vs. Mid Dose | -7.313 | -17.24 to 2.617 | 0.2606 |
|  | *Rd7* Untreated vs. High Dose | -9.951 | -19.31 to -0.5885 | 0.0321 |
|  | *Rd7* Low Dose vs. Mid Dose | 3.096 | -6.459 to 12.65 | 0.9527 |
|  | *Rd7* Low Dose vs. High Dose | 0.4590 | -8.505 to 9.423 | >0.9999 |
|  | *Rd7* Mid Dose vs. High Dose | -2.637 | -12.57 to 7.293 | 0.9837 |

**Table S7. Multiple comparisons for 6M post *NR2E3* treated *rd7* scotopic b-wave amplitude.**

| Group | Tukey's multiple comparisons test | Mean Diff. | 95.00% CI of diff. | Adjusted P Value |
| --- | --- | --- | --- | --- |
| 1M treated 1M post | B6 vs *rd7* untreated | 238.5 | 93.02 to 384.0 | 0.0002 |
|  | B6 vs *rd7* Low Dose | 217.8 | 60.68 to 374.9 | 0.0020 |
|  | B6 vs *rd7* Mid Dose | 203.4 | 69.42 to 337.4 | 0.0006 |
|  | B6 vs *rd7* High Dose | 255.3 | 113.7 to 396.9 | <0.0001 |
|  | *Rd7* Untreated vs. Low Dose | -20.69 | -166.2 to 124.8 | 0.9998 |
|  | *Rd7* Untreated vs. Mid Dose | -35.07 | -155.2 to 85.04 | 0.9784 |
|  | *Rd7* Untreated vs. High Dose | 16.83 | -111.7 to 145.4 | 0.9999 |
|  | *Rd7* Low Dose vs. Mid Dose | -14.38 | -148.4 to 119.6 | >0.9999 |
|  | *Rd7* Low Dose vs. High Dose | 37.52 | -104.1 to 179.1 | 0.9876 |
|  | *Rd7* Mid Dose vs. High Dose | 51.91 | -63.53 to 167.3 | 0.8221 |
|  |  |  |  |  |
| 3M treated 1M post | B6 vs *rd7* untreated | 262.8 | 213.0 to 312.7 | <0.0001 |
|  | B6 vs *rd7* Low Dose | 240.4 | 184.9 to 296.0 | <0.0001 |
|  | B6 vs *rd7* Mid Dose | 227.7 | 170.9 to 284.6 | <0.0001 |
|  | B6 vs *rd7* High Dose | 241.2 | 180.8 to 301.5 | <0.0001 |
|  | *Rd7* Untreated vs. Low Dose | -22.39 | -62.39 to 17.62 | 0.6351 |
|  | *Rd7* Untreated vs. Mid Dose | -35.10 | -76.80 to 6.595 | 0.1564 |
|  | *Rd7* Untreated vs. High Dose | -21.68 | -68.07 to 24.72 | 0.8095 |
|  | *Rd7* Low Dose vs. Mid Dose | -12.71 | -61.15 to 35.72 | 0.9899 |
|  | *Rd7* Low Dose vs. High Dose | 0.7110 | -51.82 to 53.24 | >0.9999 |
|  | *Rd7* Mid Dose vs. High Dose | 13.43 | -40.40 to 67.25 | 0.9926 |
|  |  |  |  |  |
| 1M treated 3M post | B6 vs *rd7* untreated | 262.8 | 199.3 to 326.3 | <0.0001 |
|  | B6 vs *rd7* Low Dose | 231.9 | 159.5 to 304.3 | <0.0001 |
|  | B6 vs *rd7* Mid Dose | 204.6 | 124.3 to 284.9 | <0.0001 |
|  | B6 vs *rd7* High Dose | 210.9 | 130.6 to 291.2 | <0.0001 |
|  | *Rd7* Untreated vs. Low Dose | -30.94 | -84.06 to 22.18 | 0.5807 |
|  | *Rd7* Untreated vs. Mid Dose | -58.25 | -121.7 to 5.237 | 0.0922 |
|  | *Rd7* Untreated vs. High Dose | -51.91 | -115.4 to 11.58 | 0.1800 |
|  | *Rd7* Low Dose vs. Mid Dose | -27.32 | -99.71 to 45.07 | 0.9241 |
|  | *Rd7* Low Dose vs. High Dose | -20.98 | -93.37 to 51.41 | 0.9812 |
|  | *Rd7* Mid Dose vs. High Dose | 6.340 | -73.97 to 86.65 | >0.9999 |
|  |  |  |  |  |
| 3M treated 3M post | B6 vs *rd7* untreated | 302.8 | 197.5 to 408.1 | <0.0001 |
|  | B6 vs *rd7* Low Dose | 233.7 | 108.7 to 358.8 | <0.0001 |
|  | B6 vs *rd7* Mid Dose | 196.0 | 70.97 to 321.1 | 0.0005 |
|  | B6 vs *rd7* High Dose | 224.9 | 99.83 to 350.0 | <0.0001 |
|  | *Rd7* Untreated vs. Low Dose | -69.08 | -174.3 to 36.18 | 0.4094 |
|  | *Rd7* Untreated vs. Mid Dose | -106.8 | -212.0 to -1.504 | 0.0450 |
|  | *Rd7* Untreated vs. High Dose | -77.90 | -183.2 to 27.36 | 0.2689 |
|  | *Rd7* Low Dose vs. Mid Dose | -37.68 | -162.7 to 87.39 | 0.9723 |
|  | *Rd7* Low Dose vs. High Dose | -8.820 | -133.9 to 116.2 | >0.9999 |
|  | *Rd7* Mid Dose vs. High Dose | 28.86 | -96.21 to 153.9 | 0.9940 |
|  |  |  |  |  |
| 1M treated 6M post | B6 vs *rd7* untreated | 302.8 | 222.8 to 382.8 | <0.0001 |
|  | B6 vs *rd7* Low Dose | 198.4 | 107.3 to 289.4 | <0.0001 |
|  | B6 vs *rd7* Mid Dose | 145.5 | 50.43 to 240.7 | 0.0006 |
|  | B6 vs *rd7* High Dose | 208.1 | 113.0 to 303.2 | <0.0001 |
|  | *Rd7* Untreated vs. Low Dose | -104.4 | -179.6 to -29.23 | 0.0022 |
|  | *Rd7* Untreated vs. Mid Dose | -157.3 | -237.3 to -77.21 | <0.0001 |
|  | *Rd7* Untreated vs. High Dose | -94.68 | -174.7 to -14.63 | 0.0121 |
|  | *Rd7* Low Dose vs. Mid Dose | -52.84 | -143.9 to 38.22 | 0.5637 |
|  | *Rd7* Low Dose vs. High Dose | 9.743 | -81.32 to 100.8 | >0.9999 |
|  | *Rd7* Mid Dose vs. High Dose | 62.58 | -32.53 to 157.7 | 0.4089 |
|  |  |  |  |  |
| 3M treated 6M post | B6 vs *rd7* untreated | 324.1 | 235.7 to 412.4 | <0.0001 |
|  | B6 vs *rd7* Low Dose | 226.1 | 137.8 to 314.4 | <0.0001 |
|  | B6 vs *rd7* Mid Dose | 132.6 | 44.29 to 221.0 | 0.0013 |
|  | B6 vs *rd7* High Dose | 295.1 | 206.8 to 383.5 | <0.0001 |
|  | *Rd7* Untreated vs. Low Dose | -97.96 | -186.3 to -9.615 | 0.0233 |
|  | *Rd7* Untreated vs. Mid Dose | -191.4 | -279.8 to -103.1 | <0.0001 |
|  | *Rd7* Untreated vs. High Dose | -28.92 | -117.3 to 59.43 | 0.9486 |
|  | *Rd7* Low Dose vs. Mid Dose | -93.46 | -181.8 to -5.115 | 0.0334 |
|  | *Rd7* Low Dose vs. High Dose | 69.04 | -19.31 to 157.4 | 0.2007 |
|  | *Rd7* Mid Dose vs. High Dose | 162.5 | 74.15 to 250.8 | 0.0001 |

**Table S8. Multiple comparisons for 6M post *NR2E3* treated *rd7* scotopic a-wave amplitude.**

| Group | Tukey's multiple comparisons test | Mean Diff. | 95.00% CI of diff. | Adjusted P Value |
| --- | --- | --- | --- | --- |
| 1M treated 1M post | B6 vs *rd7* untreated | -92.79 | -132.6 to -52.96 | <0.0001 |
|  | B6 vs *rd7* Low Dose | -88.33 | -135.6 to -41.06 | <0.0001 |
|  | B6 vs *rd7* Mid Dose | -86.91 | -131.9 to -41.88 | <0.0001 |
|  | B6 vs *rd7* High Dose | -86.24 | -128.7 to -43.75 | <0.0001 |
|  | *Rd7* Untreated vs. Low Dose | 4.460 | -30.39 to 39.31 | >0.9999 |
|  | *Rd7* Untreated vs. Mid Dose | 5.882 | -25.86 to 37.63 | 0.9989 |
|  | *Rd7* Untreated vs. High Dose | 6.555 | -21.46 to 34.57 | 0.9953 |
|  | *Rd7* Low Dose vs. Mid Dose | 1.421 | -39.27 to 42.11 | >0.9999 |
|  | *Rd7* Low Dose vs. High Dose | 2.094 | -35.76 to 39.94 | >0.9999 |
|  | *Rd7* Mid Dose vs. High Dose | 0.6729 | -34.34 to 35.68 | >0.9999 |
|  |  |  |  |  |
| 3M treated 1M post | B6 vs *rd7* untreated | -101.3 | -126.8 to -75.86 | <0.0001 |
|  | B6 vs *rd7* Low Dose | -89.40 | -118.0 to -60.85 | <0.0001 |
|  | B6 vs *rd7* Mid Dose | -85.80 | -113.2 to -58.36 | <0.0001 |
|  | B6 vs *rd7* High Dose | -92.94 | -123.5 to -62.41 | <0.0001 |
|  | *Rd7* Untreated vs. Low Dose | 11.91 | -7.488 to 31.31 | 0.5349 |
|  | *Rd7* Untreated vs. Mid Dose | 15.52 | -2.193 to 33.22 | 0.1269 |
|  | *Rd7* Untreated vs. High Dose | 8.374 | -13.83 to 30.57 | 0.9322 |
|  | *Rd7* Low Dose vs. Mid Dose | 3.605 | -18.32 to 25.53 | 0.9995 |
|  | *Rd7* Low Dose vs. High Dose | -3.537 | -29.23 to 22.15 | 0.9999 |
|  | *Rd7* Mid Dose vs. High Dose | -7.142 | -31.58 to 17.30 | 0.9828 |
|  |  |  |  |  |
| 1M treated 3M post | B6 vs *rd7* untreated | -101.3 | -133.8 to -68.80 | <0.0001 |
|  | B6 vs *rd7* Low Dose | -98.81 | -136.8 to -60.85 | <0.0001 |
|  | B6 vs *rd7* Mid Dose | -91.05 | -130.0 to -52.06 | <0.0001 |
|  | B6 vs *rd7* High Dose | -82.24 | -117.7 to -46.80 | <0.0001 |
|  | *Rd7* Untreated vs. Low Dose | 2.506 | -24.41 to 29.42 | >0.9999 |
|  | *Rd7* Untreated vs. Mid Dose | 10.27 | -18.08 to 38.62 | 0.9440 |
|  | *Rd7* Untreated vs. High Dose | 19.08 | -4.160 to 42.31 | 0.1825 |
|  | *Rd7* Low Dose vs. Mid Dose | 7.762 | -26.70 to 42.22 | 0.9963 |
|  | *Rd7* Low Dose vs. High Dose | 16.57 | -13.82 to 46.96 | 0.6744 |
|  | *Rd7* Mid Dose vs. High Dose | 8.807 | -22.86 to 40.47 | 0.9869 |
|  |  |  |  |  |
| 3M treated 3M post | B6 vs *rd7* untreated | -112.0 | -146.9 to -77.08 | <0.0001 |
|  | B6 vs *rd7* Low Dose | -103.2 | -143.9 to -62.56 | <0.0001 |
|  | B6 vs *rd7* Mid Dose | -97.14 | -139.2 to -55.11 | <0.0001 |
|  | B6 vs *rd7* High Dose | -102.3 | -140.3 to -64.26 | <0.0001 |
|  | *Rd7* Untreated vs. Low Dose | 8.766 | -21.93 to 39.46 | 0.9834 |
|  | *Rd7* Untreated vs. Mid Dose | 14.83 | -17.68 to 47.34 | 0.8263 |
|  | *Rd7* Untreated vs. High Dose | 9.689 | -17.43 to 36.81 | 0.9441 |
|  | *Rd7* Low Dose vs. Mid Dose | 6.063 | -32.56 to 44.69 | 0.9996 |
|  | *Rd7* Low Dose vs. High Dose | 0.9230 | -33.29 to 35.13 | >0.9999 |
|  | *Rd7* Mid Dose vs. High Dose | -5.140 | -40.99 to 30.71 | 0.9998 |
|  |  |  |  |  |
| 1M treated 6M post | B6 vs *rd7* untreated | -112.0 | -145.4 to -78.56 | <0.0001 |
|  | B6 vs *rd7* Low Dose | -88.97 | -129.2 to -48.72 | <0.0001 |
|  | B6 vs *rd7* Mid Dose | -71.57 | -113.6 to -29.53 | <0.0001 |
|  | B6 vs *rd7* High Dose | -92.77 | -133.0 to -52.52 | <0.0001 |
|  | *Rd7* Untreated vs. Low Dose | 23.01 | -8.120 to 54.14 | 0.2831 |
|  | *Rd7* Untreated vs. Mid Dose | 40.40 | 6.994 to 73.81 | 0.0089 |
|  | *Rd7* Untreated vs. High Dose | 19.20 | -11.93 to 50.33 | 0.5063 |
|  | *Rd7* Low Dose vs. Mid Dose | 17.40 | -22.85 to 57.65 | 0.8559 |
|  | *Rd7* Low Dose vs. High Dose | -3.805 | -42.18 to 34.57 | >0.9999 |
|  | *Rd7* Mid Dose vs. High Dose | -21.20 | -61.45 to 19.05 | 0.6910 |
|  |  |  |  |  |
| 3M treated 6M post | B6 vs *rd7* untreated | -118.3 | -163.3 to -73.27 | <0.0001 |
|  | B6 vs *rd7* Low Dose | -98.95 | -148.7 to -49.21 | <0.0001 |
|  | B6 vs *rd7* Mid Dose | -55.67 | -107.6 to -3.719 | 0.0294 |
|  | B6 vs *rd7* High Dose | -117.0 | -169.0 to -65.07 | <0.0001 |
|  | *Rd7* Untreated vs. Low Dose | 19.31 | -23.11 to 61.73 | 0.8019 |
|  | *Rd7* Untreated vs. Mid Dose | 62.59 | 17.60 to 107.6 | 0.0023 |
|  | *Rd7* Untreated vs. High Dose | 1.237 | -43.76 to 46.23 | >0.9999 |
|  | *Rd7* Low Dose vs. Mid Dose | 43.28 | -6.462 to 93.02 | 0.1225 |
|  | *Rd7* Low Dose vs. High Dose | -18.07 | -67.81 to 31.67 | 0.9262 |
|  | *Rd7* Mid Dose vs. High Dose | -61.35 | -113.3 to -9.399 | 0.0126 |

**Table S9. Multiple comparisons for 6M post *NR2E3* treated *rd7* photopic b-wave amplitude.**

| Group | Tukey's multiple comparisons test | Mean Diff. | 95.00% CI of diff. | Adjusted P Value |
| --- | --- | --- | --- | --- |
| 1M treated 1M post | B6 vs *rd7* untreated | 42.08 | 0.9872 to 83.17 | 0.0415 |
|  | B6 vs *rd7* Low Dose | 41.19 | -9.137 to 91.52 | 0.1779 |
|  | B6 vs *rd7* Mid Dose | 41.19 | -9.139 to 91.51 | 0.1780 |
|  | B6 vs *rd7* High Dose | 45.57 | 2.648 to 88.49 | 0.0308 |
|  | *Rd7* Untreated vs. Low Dose | -0.8890 | -41.98 to 40.20 | >0.9999 |
|  | *Rd7* Untreated vs. Mid Dose | -0.8910 | -41.98 to 40.20 | >0.9999 |
|  | *Rd7* Untreated vs. High Dose | 3.488 | -28.10 to 35.08 | >0.9999 |
|  | *Rd7* Low Dose vs. Mid Dose | -0.002000 | -50.33 to 50.32 | >0.9999 |
|  | *Rd7* Low Dose vs. High Dose | 4.377 | -38.54 to 47.30 | >0.9999 |
|  | *Rd7* Mid Dose vs. High Dose | 4.379 | -38.54 to 47.30 | >0.9999 |
|  |  |  |  |  |
| 3M treated 1M post | B6 vs *rd7* untreated | 38.82 | 4.717 to 72.93 | 0.0152 |
|  | B6 vs *rd7* Low Dose | 45.57 | 8.016 to 83.12 | 0.0075 |
|  | B6 vs *rd7* Mid Dose | 37.15 | 1.199 to 73.10 | 0.0380 |
|  | B6 vs *rd7* High Dose | 48.69 | 7.928 to 89.46 | 0.0090 |
|  | *Rd7* Untreated vs. Low Dose | 6.743 | -18.45 to 31.93 | 0.9898 |
|  | *Rd7* Untreated vs. Mid Dose | -1.672 | -24.41 to 21.07 | >0.9999 |
|  | *Rd7* Untreated vs. High Dose | 9.870 | -19.90 to 39.64 | 0.9657 |
|  | *Rd7* Low Dose vs. Mid Dose | -8.415 | -36.05 to 19.22 | 0.9785 |
|  | *Rd7* Low Dose vs. High Dose | 3.127 | -30.53 to 36.79 | >0.9999 |
|  | *Rd7* Mid Dose vs. High Dose | 11.54 | -20.33 to 43.41 | 0.9453 |
|  |  |  |  |  |
| 1M treated 3M post | B6 vs *rd7* untreated | 38.82 | 2.112 to 75.54 | 0.0313 |
|  | B6 vs *rd7* Low Dose | 45.15 | -2.241 to 92.55 | 0.0721 |
|  | B6 vs *rd7* Mid Dose | 42.52 | -2.854 to 87.90 | 0.0811 |
|  | B6 vs *rd7* High Dose | 25.51 | -14.38 to 65.39 | 0.4761 |
|  | *Rd7* Untreated vs. Low Dose | 6.330 | -30.38 to 43.04 | 0.9993 |
|  | *Rd7* Untreated vs. Mid Dose | 3.700 | -30.37 to 37.77 | >0.9999 |
|  | *Rd7* Untreated vs. High Dose | -13.32 | -39.64 to 13.00 | 0.7459 |
|  | *Rd7* Low Dose vs. Mid Dose | -2.631 | -48.01 to 42.75 | >0.9999 |
|  | *Rd7* Low Dose vs. High Dose | -19.65 | -59.54 to 20.24 | 0.7705 |
|  | *Rd7* Mid Dose vs. High Dose | -17.02 | -54.49 to 20.45 | 0.8345 |
|  |  |  |  |  |
| 3M treated 3M post | B6 vs *rd7* untreated | 59.89 | 19.29 to 100.5 | 0.0007 |
|  | B6 vs *rd7* Low Dose | 51.46 | 5.055 to 97.86 | 0.0207 |
|  | B6 vs *rd7* Mid Dose | 43.31 | -3.092 to 89.71 | 0.0826 |
|  | B6 vs *rd7* High Dose | 56.51 | 13.10 to 99.91 | 0.0037 |
|  | *Rd7* Untreated vs. Low Dose | -8.438 | -44.35 to 27.47 | 0.9946 |
|  | *Rd7* Untreated vs. Mid Dose | -16.59 | -52.50 to 19.33 | 0.8151 |
|  | *Rd7* Untreated vs. High Dose | -3.386 | -35.33 to 28.56 | >0.9999 |
|  | *Rd7* Low Dose vs. Mid Dose | -8.147 | -50.51 to 34.21 | 0.9985 |
|  | *Rd7* Low Dose vs. High Dose | 5.052 | -34.00 to 44.10 | 0.9999 |
|  | *Rd7* Mid Dose vs. High Dose | 13.20 | -25.85 to 52.25 | 0.9573 |
|  |  |  |  |  |
| 1M treated 6M post | B6 vs *rd7* untreated | 59.89 | 25.97 to 93.81 | <0.0001 |
|  | B6 vs *rd7* Low Dose | 28.02 | -13.85 to 69.89 | 0.3966 |
|  | B6 vs *rd7* Mid Dose | 11.26 | -30.61 to 53.13 | 0.9867 |
|  | B6 vs *rd7* High Dose | 29.77 | -10.32 to 69.86 | 0.2729 |
|  | *Rd7* Untreated vs. Low Dose | -31.87 | -65.79 to 2.047 | 0.0779 |
|  | *Rd7* Untreated vs. Mid Dose | -48.63 | -82.55 to -14.71 | 0.0013 |
|  | *Rd7* Untreated vs. High Dose | -30.12 | -61.81 to 1.574 | 0.0721 |
|  | *Rd7* Low Dose vs. Mid Dose | -16.76 | -58.63 to 25.11 | 0.8935 |
|  | *Rd7* Low Dose vs. High Dose | 1.753 | -38.34 to 41.84 | >0.9999 |
|  | *Rd7* Mid Dose vs. High Dose | 18.51 | -21.58 to 58.60 | 0.8039 |
|  |  |  |  |  |
| 3M treated 6M post | B6 vs *rd7* untreated | 68.82 | 22.08 to 115.6 | 0.0012 |
|  | B6 vs *rd7* Low Dose | 39.67 | -14.29 to 93.64 | 0.2722 |
|  | B6 vs *rd7* Mid Dose | 9.960 | -44.01 to 63.93 | 0.9984 |
|  | B6 vs *rd7* High Dose | 80.75 | 26.79 to 134.7 | 0.0010 |
|  | *Rd7* Untreated vs. Low Dose | -29.14 | -75.88 to 17.59 | 0.4664 |
|  | *Rd7* Untreated vs. Mid Dose | -58.86 | -105.6 to -12.12 | 0.0068 |
|  | *Rd7* Untreated vs. High Dose | 11.94 | -34.80 to 58.67 | 0.9885 |
|  | *Rd7* Low Dose vs. Mid Dose | -29.71 | -83.68 to 24.25 | 0.6151 |
|  | *Rd7* Low Dose vs. High Dose | 41.08 | -12.89 to 95.05 | 0.2358 |
|  | *Rd7* Mid Dose vs. High Dose | 70.79 | 16.83 to 124.8 | 0.0045 |

**Table S10. Multiple comparisons for 6M post *NR2E3* treated *rd7* photopic a-wave amplitude.**

| Group | Tukey's multiple comparisons test | Mean Diff. | 95.00% CI of diff. | Adjusted P Value |
| --- | --- | --- | --- | --- |
| 1M treated 1M post | B6 vs *rd7* untreated | -7.588 | -12.77 to -2.400 | 0.0007 |
|  | B6 vs *rd7* Low Dose | -7.412 | -13.82 to -1.009 | 0.0133 |
|  | B6 vs *rd7* Mid Dose | -7.474 | -13.40 to -1.545 | 0.0052 |
|  | B6 vs *rd7* High Dose | -7.644 | -12.79 to -2.493 | 0.0006 |
|  | *Rd7* Untreated vs. Low Dose | 0.1753 | -5.012 to 5.363 | >0.9999 |
|  | *Rd7* Untreated vs. Mid Dose | 0.1141 | -4.474 to 4.702 | >0.9999 |
|  | *Rd7* Untreated vs. High Dose | -0.05591 | -3.582 to 3.471 | >0.9999 |
|  | *Rd7* Low Dose vs. Mid Dose | -0.06120 | -5.990 to 5.867 | >0.9999 |
|  | *Rd7* Low Dose vs. High Dose | -0.2312 | -5.382 to 4.920 | >0.9999 |
|  | *Rd7* Mid Dose vs. High Dose | -0.1700 | -4.717 to 4.377 | >0.9999 |
|  |  |  |  |  |
| 3M treated 1M post | B6 vs *rd7* untreated | -8.852 | -12.78 to -4.925 | <0.0001 |
|  | B6 vs *rd7* Low Dose | -7.224 | -11.44 to -3.005 | <0.0001 |
|  | B6 vs *rd7* Mid Dose | -8.238 | -12.41 to -4.067 | <0.0001 |
|  | B6 vs *rd7* High Dose | -6.742 | -11.38 to -2.101 | 0.0007 |
|  | *Rd7* Untreated vs. Low Dose | 1.628 | -1.217 to 4.473 | 0.6200 |
|  | *Rd7* Untreated vs. Mid Dose | 0.6141 | -2.159 to 3.387 | 0.9967 |
|  | *Rd7* Untreated vs. High Dose | 2.110 | -1.330 to 5.550 | 0.5346 |
|  | *Rd7* Low Dose vs. Mid Dose | -1.014 | -4.187 to 2.159 | 0.9714 |
|  | *Rd7* Low Dose vs. High Dose | 0.4820 | -3.288 to 4.252 | >0.9999 |
|  | *Rd7* Mid Dose vs. High Dose | 1.496 | -2.220 to 5.212 | 0.9062 |
|  |  |  |  |  |
| 1M treated 3M post | B6 vs *rd7* untreated | -8.852 | -13.17 to -4.533 | <0.0001 |
|  | B6 vs *rd7* Low Dose | -8.979 | -13.84 to -4.116 | <0.0001 |
|  | B6 vs *rd7* Mid Dose | -5.933 | -11.04 to -0.8283 | 0.0125 |
|  | B6 vs *rd7* High Dose | -8.621 | -13.26 to -3.980 | <0.0001 |
|  | *Rd7* Untreated vs. Low Dose | -0.1261 | -3.576 to 3.323 | >0.9999 |
|  | *Rd7* Untreated vs. Mid Dose | 2.920 | -0.8636 to 6.703 | 0.2450 |
|  | *Rd7* Untreated vs. High Dose | 0.2316 | -2.897 to 3.360 | >0.9999 |
|  | *Rd7* Low Dose vs. Mid Dose | 3.046 | -1.348 to 7.439 | 0.3736 |
|  | *Rd7* Low Dose vs. High Dose | 0.3577 | -3.486 to 4.202 | >0.9999 |
|  | *Rd7* Mid Dose vs. High Dose | -2.688 | -6.834 to 1.458 | 0.4600 |
|  |  |  |  |  |
| 3M treated 3M post | B6 vs *rd7* untreated | -10.22 | -14.44 to -5.995 | <0.0001 |
|  | B6 vs *rd7* Low Dose | -7.049 | -11.97 to -2.129 | 0.0011 |
|  | B6 vs *rd7* Mid Dose | -6.817 | -11.74 to -1.898 | 0.0017 |
|  | B6 vs *rd7* High Dose | -8.399 | -13.49 to -3.311 | 0.0001 |
|  | *Rd7* Untreated vs. Low Dose | 3.169 | -0.5455 to 6.884 | 0.1438 |
|  | *Rd7* Untreated vs. Mid Dose | 3.401 | -0.3138 to 7.116 | 0.0937 |
|  | *Rd7* Untreated vs. High Dose | 1.820 | -2.115 to 5.754 | 0.8129 |
|  | *Rd7* Low Dose vs. Mid Dose | 0.2317 | -4.259 to 4.723 | >0.9999 |
|  | *Rd7* Low Dose vs. High Dose | -1.350 | -6.024 to 3.325 | 0.9818 |
|  | *Rd7* Mid Dose vs. High Dose | -1.581 | -6.255 to 3.093 | 0.9567 |
|  |  |  |  |  |
| 1M treated 6M post | B6 vs *rd7* untreated | -10.22 | -15.44 to -4.998 | <0.0001 |
|  | B6 vs *rd7* Low Dose | -5.933 | -12.50 to 0.6361 | 0.1014 |
|  | B6 vs *rd7* Mid Dose | -8.860 | -15.43 to -2.291 | 0.0026 |
|  | B6 vs *rd7* High Dose | -7.148 | -13.44 to -0.8585 | 0.0167 |
|  | *Rd7* Untreated vs. Low Dose | 4.285 | -0.9351 to 9.506 | 0.1746 |
|  | *Rd7* Untreated vs. Mid Dose | 1.358 | -3.862 to 6.579 | 0.9895 |
|  | *Rd7* Untreated vs. High Dose | 3.070 | -1.793 to 7.934 | 0.4754 |
|  | *Rd7* Low Dose vs. Mid Dose | -2.927 | -9.496 to 3.642 | 0.8347 |
|  | *Rd7* Low Dose vs. High Dose | -1.215 | -7.504 to 5.074 | 0.9983 |
|  | *Rd7* Mid Dose vs. High Dose | 1.712 | -4.577 to 8.001 | 0.9863 |
|  |  |  |  |  |
| 3M treated 6M post | B6 vs *rd7* untreated | -10.77 | -19.34 to -2.203 | 0.0077 |
|  | B6 vs *rd7* Low Dose | -6.644 | -14.85 to 1.558 | 0.1717 |
|  | B6 vs *rd7* Mid Dose | -8.677 | -17.24 to -0.1104 | 0.0457 |
|  | B6 vs *rd7* High Dose | -4.863 | -13.43 to 3.704 | 0.5629 |
|  | *Rd7* Untreated vs. Low Dose | 4.125 | -4.077 to 12.33 | 0.6950 |
|  | *Rd7* Untreated vs. Mid Dose | 2.092 | -6.474 to 10.66 | 0.9899 |
|  | *Rd7* Untreated vs. High Dose | 5.907 | -2.660 to 14.47 | 0.3322 |
|  | *Rd7* Low Dose vs. Mid Dose | -2.033 | -10.24 to 6.169 | 0.9890 |
|  | *Rd7* Low Dose vs. High Dose | 1.781 | -6.421 to 9.983 | 0.9950 |
|  | *Rd7* Mid Dose vs. High Dose | 3.814 | -4.753 to 12.38 | 0.8025 |
